# Parental democratic communication and adolescent well-being in an era of loneliness: the mediating role of societal trust

**DOI:** 10.3389/fpsyt.2024.1500937

**Published:** 2024-11-28

**Authors:** Simin Liu, Sydney X. Hu, Lanxin Su

**Affiliations:** ^1^ Kiang Wu Nursing College of Macau, Macao, Macao SAR, China; ^2^ Medicine School of Hunan Normal University, Changsha, China

**Keywords:** trust-mediated well-being, parental democratic communication, loneliness, Chinese adolescents, multi-group SEM

## Abstract

**Background:**

In an era marked by increasing loneliness, understanding the impact of parenting practices on adolescent well-being and resilience is crucial. This study investigates the relationship between parental democratic communication and key indicators of adolescent adjustment and well-being in China, with a focus on the mediating role of societal trust.

**Objective:**

The study aimed to examine the direct effects of parental democratic communication on Chinese adolescents’ subjective well-being and to explore the mediating roles of societal trust in this relationship.

**Methods:**

Data were collected from 691 high school students as part of the 2020 Chinese Family Panel Studies (CFPS). The sample was divided into two age groups: 16-17 years old (n=493) and 18 years old (n=198). Multi-group Structural Equation Modeling (SEM) was used to analyze the data.

**Results:**

SEM analysis revealed age-specific effects of parental democratic communication (PDC) on subjective well-being (SWB). For ages 16-17, PDC directly influenced SWB (β=0.269, p<0.001) with significant serial mediations through societal trust, negative emotion, and pleasant life experiences. For 18-year-olds, only societal trust mediated the PDC-SWB relationship (β=0.16, p<0.01). Meanwhile, the effect of societal trust is superior to that of other mediating variables in both groups. Multi-group analysis showed measurement invariance but differences in structural relationships across age groups.

**Conclusions:**

Parental democratic communication has a direct as well as serial mediated impact on mid-adolescents’ subjective well-being and an indirect impact through societal trust in late adolescence, among Chinese adolescents. These results point to a pattern we term “Societal Trust-Mediated Well-Being,” which appears to wield greater influence than negative emotions or pleasant life experiences, particularly among older adolescents. These results underscore the need for developmentally tailored approaches and integrative interventions that adapt to the changing dynamics of adolescent well-being in a rapidly evolving society.

## 1 Introduction

Adolescents in the 21st century confront an array of unique challenges as they navigate a world profoundly transformed by technological advancements, globalization, and evolving social norms (1). These societal shifts have prompted researchers to reassess the factors that contribute to adolescent well-being and resilience (2). Recent studies have revealed troubling trends in adolescent mental health. Twenge et al. (3) documented a persistent decline in life satisfaction and a corresponding increase in mental distress among adolescents between 2005 and 2017. In a similar vein, Cosma et al. (4) observed a downward trajectory in adolescent mental well-being across 24 European countries from 2014 to 2018.

From the perspective of positive psychology, subjective well-being serves multiple functional roles in adolescent development. A longitudinal investigation by Rose et al. (5) demonstrated that higher life satisfaction in adolescents predicted enhanced academic performance, fewer behavioral issues, and improved social relationships over time. Furthermore, subjective well-being functions as a psychological resource, enhancing resilience and aiding adolescents in coping with life stressors and challenges (6, 7). In light of ongoing global challenges, including the COVID-19 pandemic, Marques de Miranda et al. (8) underscored the critical importance of understanding and promoting factors that contribute to adolescent well-being.

Contemporary social trust faces numerous challenges, including burnout (9, 10), misinformation (11), income inequality (12), and environments that foster loneliness, characterized by social isolation and over-reliance on social media (13). The COVID-19 pandemic has exacerbated these issues, leading to increased social isolation and disrupting normal social development processes (14). Among these factors, parenting practices play a crucial role in shaping adolescent outcomes, as the family context remains a primary influence during this formative period (15). Positive familial relationships and social connections have been consistently linked to better mental health outcomes (16).

Parental democratic communication, characterized by open dialogue, emotional support, and respect for autonomy, has been linked to positive youth development, particularly in Western studies (1). This approach encourages adolescents’ participation in family decision-making and promotes mutual respect between parents and children (17). Key elements include open two-way communication, shared decision-making, parental understanding in interpreting rules, and respect for adolescent autonomy (18, 19). Research suggests that democratic communication reduces anxiety, depression and internalization behaviors (20) while increasing self-esteem and social adjustment in adolescents (21). Effective parent-child communication fosters family cohesion and supports pleasant life experiences, which serve as a psychological buffer against negative emotions and enhance adolescents’ resilience and well-being (22–28). However, studies in non-Western settings, particularly in China, are limited and show conflicting results (29–32). This gap is concerning given China’s unique cultural context and rapid societal changes.

Societal trust, a key component of social capital, plays a crucial role in adolescent psychosocial development (33). For adolescents, it encompasses interpersonal trust, trust in institutions, and a sense of social connectedness (34, 35). Parental democratic communication is thought to foster societal trust by shaping beliefs about social justice and others’ dependability (36–38). While higher levels of societal trust have been linked to increased subjective well-being in adolescents (39–42), the potential mediating role of social trust between parental democratic communication and adolescent well-being remains underexplored.

Adolescence encompasses distinct developmental stages, with significant changes occurring between early/middle (16-17 years) and late adolescence/emerging adulthood (18 years) (43–45). The age of 18 marks a critical transition, legally defined as adulthood and characterized by new responsibilities and autonomy. Developmentally, 18-year-olds face unique challenges in identity formation and relationship establishment (46), supported by ongoing prefrontal cortex maturation that influences emotional regulation and decision-making (47). This age often coincides with major life changes like high school graduation or workforce entry, described as an “experience of loss” affecting mental health (48). Importantly, research demonstrates age-specific effects of parenting on adolescent outcomes: parental control’s impact on depressive symptoms decreases by late adolescence (49), while the influence of parental communication and warmth varies between 16-17 and 18-year-olds (50, 51). These developmental, neurological, social, and familial differences justify separate examination of 16-17 and 18-year-old cohorts to capture their distinct experiences and needs.

**Figure 1:**
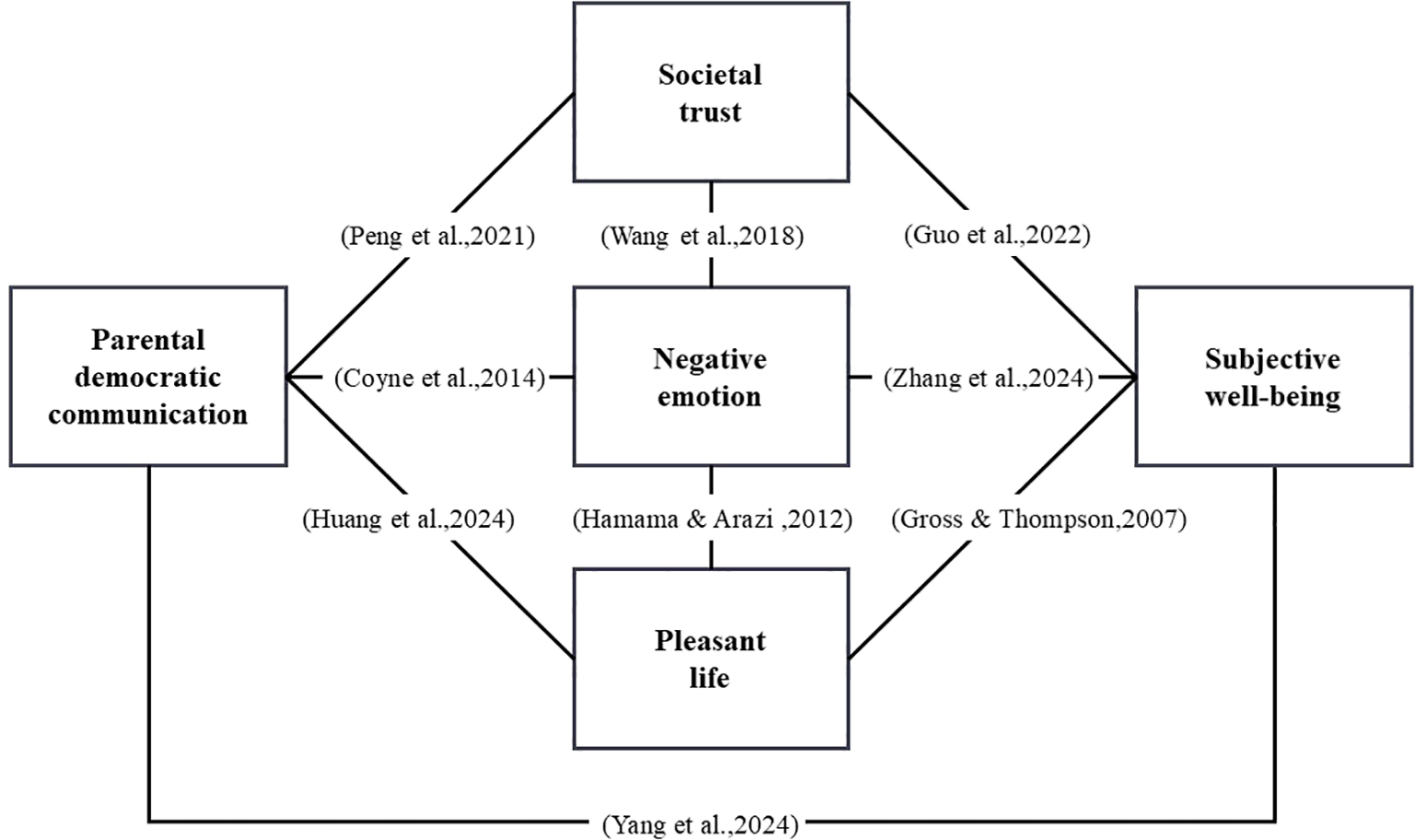
Conceptual framework (52).

While bivariate correlations have been well established among parental democratic communication, societal trust, negative emotions, pleasant life experiences, and adolescent well-being (**Figure 1**), gaps remain in understanding the directions and magnitudes of these relationships. Particularly in non-Western contexts, the specific mediating role of social trust between democratic parental communication and adolescent well-being remains underexplored, as does the variation of this relationship across different stages of adolescence.

Using multi-group Structural Equation Modeling (SEM) to test a complex theoretical model comprehensively, this study aims to examine the effects of parental democratic communication on adolescents’ subjective well-being, focusing on the mediating role of societal trust. SEM allows for the accounting of measurement error and enables rigorous comparison across age groups. Specifically, this investigation seeks to address the following research questions:

1) How does parental democratic communication influence subjective well-being among Chinese adolescents?

2) To what extent does societal trust mediate the relationship between parental democratic communication and adolescents’ subjective well-being?

3) How do the effects of parental democratic communication on adolescent well-being differ between mid-adolescents (16-17 years old) and late adolescents (18 years old)?

By addressing these questions, this study aims to contribute to a broader understanding of parental communication and societal trust in adolescent development, providing insights specific to the Chinese cultural context. This research will enhance our theoretical understanding of the mechanisms through which parental communication influences adolescent well-being and offer practical implications for supporting age-sensitive adolescent development in rapidly changing societies.

## 2 Materials and methods

### 2.1 Participants and procedure

This investigation drew upon data from the 2020 China Family Panel Studies (CFPS), a nationally representative survey funded by the National Natural Science Foundation of China and administered by Peking University’s Institute of Social Science Survey (53). Our sample selection process was as follows:

Inclusion criteria:

1. Age between 16-18 years old

Exclusion criteria:

1. Missing data for important variables or total missing data for a respondent reaching 20% or more

From the initial 28530 samples, we selected respondents aged 16-18 years (n=910). We excluded cases with missing data for key variables or where total missing data exceeded 20% per respondent. The final sample consisted of 691respondents (493 aged 16-17 and 198 aged 18), with balanced distribution of gender and urban/rural residency.

### 2.2 Measures

Parental Democratic Communication. We assessed this construct based on Baumrind’s (54) theory of democratic parenting using six items that measured the frequency of parents’ democratic communication styles over the preceding 12 months. Including “Parents ask for reasons”, “Parents encourage you to try to do things”, “Parents talk to you kindly”, “Parents encourage you to think independently”, “Parents tell you why” and “Parents like to talk to you.” Participants responded on a 5-point Likert scale ranging from 1 (never) to 5 (always), with higher scores reflecting stronger democratic parental communication. The scale demonstrated good internal consistency (Cronbach’s α = .836 for 16-17-year-olds;. 809 for 18-year-olds).

Societal Trust. With reference to the key dimensions of social trust identified in previous research (55), and based on the results of principal component factor analysis, we selected three items - trust in neighbors, local government officials, and doctors - as measures of societal trust. These items best reflect social trust in everyday life.^1^ This construct was measured using three items assessing trust in neighbors, local government officials, and doctors. Responses were recorded on a 10-point scale from 0 (very distrustful) to 10 (very trusting), with higher scores indicating greater community trust. The internal consistency of the three-item societal trust measure was assessed using Cronbach’s alpha. For 16-17-year-olds, α = .661, and for 18-year-olds, α = .620 (57). This is consistent with findings on the multidimensional nature of social trust in China (58).

Negative Emotion and Pleasant Life. The CFPS2020 used a modified version of the Center for Epidemiologic Studies Depression Scale (CESD-8) (59). Based on its core characteristics (60) and supported by factor analysis, we conceptualized the scale as having two dimensions: pleasant life and negative emotions (61, 62). The Pleasant Life construct was measured using two items from the CESD-8: “I feel happy” and “I live a happy life.” Responses were recorded on the same 4-point scale as the Negative Emotions subscale, ranging from 1 (less than a day) to 4 (5-7 days). Higher scores on this subscale indicate a greater sense of life satisfaction. The two items showed moderate correlation (r = .572, p <.01 for 16-17-year-olds; r = .659, p <.01 for 18-year-olds), supporting their use as a composite measure.

To refine the negative emotions subscale, we conducted a series of analyses:

1. Factor Analysis: Our initial factor analysis supported a two-factor structure, aligning with previous research on emotion dynamics in major depressive disorder (60).

2. Item Reduction: Within the negative emotions factor, we examined the communalities of the six original items. Two items, “sleep difficulties” and “feeling life is unmanageable,” were removed due to common factor variance extraction rate below 0.5 (63), indicating they shared less variance with other items (**Supplementary Material 4**).

3. Model Refinement: Using AMOS software, we examined modification indices for the remaining items. The covariance between error terms for “I felt everything I did was an effort” and “I felt depressed” was notably high (MI = 7.83) in the 18-year-old group, suggesting potential redundancy or inconsistency with the model.

4. Final Item Selection: Considering these statistical results and aiming to improve model fit, parsimony, and explanatory power while maintaining theoretical integrity, we further removed the item “I felt everything I did was an effort.”

The final negative emotions subscale comprised three items: “I felt depressed,” “I felt lonely,” and “I felt sad.” Participants were asked to report the frequency of these negative emotions over the past week. Responses were recorded on a 4-point scale ranging from 1 (less than a day) to 4 (5-7 days), with higher scores indicating more frequent negative emotions. This refined subscale balances statistical considerations with the core theoretical construct of negative emotions in depression. The scale demonstrated good internal consistency, with Cronbach’s alpha of.744 for 16-17-year-olds and.708 for 18-year-olds, surpassing the conventional.70 threshold for acceptable reliability.

Subjective Well-being. We assessed this construct using three items that measured happiness, life satisfaction, and future confidence. Due to the mixed scale format, items were standardized prior to reliability analysis. The scale showed acceptable internal consistency (Cronbach’s α = .689 for 16-17-year-olds;.666 for 18-year-olds).

All measures were derived from the CFPS questionnaire, which has been validated for use in the Chinese context (53). We included gender and urban-rural residence as control variables.

### 2.3 Data analysis

To address the non-consistency in score statistics between questions, we standardized individual question scores before aggregating them to construct final scores for latent variables. We computed descriptive statistics using SPSS 26.0 and examined the factor structure of key variables through exploratory factor analysis (EFA).

We tested hypothesized mediation models and conducted multicohort analyses across age groups using structural equation modeling (SEM) in AMOS 26.0 (64). Following Anderson and Gerbing’s (65) two-step approach, we first estimated measurement models using confirmatory factor analysis (CFA) to assess construct validity and reliability. We then estimated the structural model to test hypothesized relationships and mediating roles of societal trust, negative emotions, and pleasant life.

We use Maximum Likelihood (ML), set the convergence criteria from 1E-05 to 0.001, and limit the number of iterations to 50, comparative fit index (CFI), Tucker-Lewis index (TLI), root mean square error of approximation (RMSEA), and standardized root mean square residual (SRMR) to evaluate model fit. We tested mediating effects using a bootstrap method (2,000 resamples) to estimate bias-corrected 95% confidence intervals (CIs), with significance determined if the 95% CI did not include zero (66). Using age as the grouping variable, we examined unconstrained, measurement-weighted, structural-weighted, structural covariance, structural residual, and measurement-residual models for the two age groups (29–31) to assess model structure homogeneity, factor loadings, intercepts, and error variances. We determined model stability and invariance by comparing the absolute value of the critical ratio (α = .05 corresponds to a critical value of 1.96) of fit metrics and parameter differences across models (67). Pathways with critical ratios > 1.96 indicated significant differences, determining pathway influence across groups (68).

To rigorously test our model’s robustness, we conducted a comprehensive sensitivity analysis (**Supplementary Material 2**). This analysis (69) involved varying several key aspects of our modeling approach: Employing different parameter estimation methods; Adjusting convergence criteria; Increasing the number of iterations; Expanding the number of bootstrap replications;and testing theoretically feasible alternative models. The results of this sensitivity analysis demonstrated remarkable consistency and stability in our model’s performance. Across these variations, the key fit indices (CFI, TLI, RMSEA, and SRMR) remained largely unchanged, exhibiting only minor fluctuations. This stability across different analytical conditions provides strong evidence for the robustness of our model, enhancing confidence in the reliability and generalizability of our findings.

In this study, semPower package 2.1.1 of R version 4.3.1 (2023-06-16 ucrt) was used for power analysis to check the reasonableness of the sample size (**Supplementary Material 3**). Calculations using the RMSEA values and degrees of freedom of both models yielded an actual efficacy of 80.03% in the 16-17-year-old group and 80.13% in the 18-year-old group, which are both greater than 80%. This result proves that the sample sizes of the two models are at a reasonable level, which can provide reliable support for the conclusions of the study and ensure that the results of the study are statistically valid and credible.

## 3 Results

### 3.1 Test of common method bias

We assessed common method bias using Harman’s single-factor method (70). For both age groups, five factors emerged with eigenvalues greater than 1. The first factor accounted for 19.958% and 18.096% of the variance for the 16-17 and 18-year-old groups, respectively. These values fall below the 40% threshold, suggesting no significant common method bias in either group.

### 3.2 Descriptive statistics and correlations

For the 16-17 age group, skewness values ranged from -0.62 to 1.23, and kurtosis values ranged from -0.25 to 2.48. The 18-year-old group exhibited skewness values between -0.63 and 0.54, and kurtosis values between -0.37 and 1.38 (**Table 1**). These values fall within acceptable ranges, indicating normal distribution of the data (71).

**Table 1:** Descriptive statistics of key variables for 16-17 and 18-year-old groups.

Group	Latent variables	M	SD	Skewness	Kurtosis	Minimum	Maximum
16-17	1.Parental democratic communication	4.05	1.01	-0.62	1.03	0.25	6.00
	2.Societal trust	1.94	0.49	-0.24	-0.15	0.22	3.00
	3.Negative emotion	0.53	0.53	1.23	2.48	0.00	3.00
	4.Pleasant life	1.47	0.45	-0.51	-0.25	0.00	2.00
	5.Subjective well-being	2.27	0.45	-0.46	0.26	0.39	3.00
18	1.Parental democratic communication	4.05	0.99	-0.63	1.38	0.00	6.00
	2.Societal trust	1.91	0.47	-0.57	0.25	0.67	3.00
	3.Negative emotion	0.56	0.49	0.54	-0.20	0.00	2.00
	4.Pleasant life	1.45	0.46	-0.43	-0.37	0.00	2.00
	5.Subjective well-being	2.19	0.41	-0.09	0.17	0.72	3.00

Correlation analyses (**Table 2**) revealed significant relationships between key variables. In both age groups, subjective well-being showed significant positive correlations with parental democratic communication (r = 0.30, p <.01 for 16-17; r = 0.25, p <.01 for 18), societal trust (r = 0.34, p <.01 for 16-17; r = 0.27, p <.01 for 18), and pleasant life (r = 0.25, p <.01 for 16-17; r = 0.14, p <.05 for 18). Conversely, subjective well-being demonstrated significant negative correlations with negative emotions (r = -0.34, p <.01 for 16-17; r = -0.23, p <.01 for 18). In the 16-17 age group, pleasant living was positively correlated with parental democratic communication (r = 0.23, p <.01) and social trust (r = 0.15, p <.01) and negatively correlated with negative emotions (r = -0.28, p <.01). In the 18-year-old group, pleasant life was positively correlated with parental democratic communication (r = 0.21, p <.01) and negatively correlated with negative emotions (r = -0.16, p <.05). Negative emotions were negatively correlated with parental democratic communication (r = -0.19, p <.01 for 16-17) and social trust (r = -0.19, p <.01 for 16-17; r = -0.14, p <.05 for 18). In both groups, social trust was positively correlated with parental democratic communication (r = 0.15, p <.01 for 16-17; r = 0.26, p <.01 for 18).

**Table 2:** Bivariate correlations among latent variables for 16-17 and 18-year-old groups.

Group	Latent variables	1	2	3	4	5
16-17	1.Parental democratic communication	1	—	—	—	—
	2. Societal trust	0.15**	1	—	—	—
	3. Negative emotion	-0.19**	-0.19**	1	—	—
	4. Pleasant life	0.23**	0.15**	-0.28**	1	—
	5. Subjective well-being	0.30**	0.34**	-0.34**	0.25**	1
18	1. Parental democratic communication	1	—	—	—	—
	2. Societal trust	0.26**	1	—	—	—
	3. Negative emotion	-0.10	-0.14*	1	—	—
	4. Pleasant life	0.21**	0.13	-0.16*	1	—
	5. Subjective well-being	0.25**	0.27**	-0.23**	0.14*	1

** p<0.01, *p<0.05.

### 3.3 Measurement model

We evaluated the measurement model using exploratory factor analysis (EFA), followed by confirmatory factor analysis (CFA). We conducted these analyses separately for both age groups.

#### 3.3.1 Exploratory factor analysis (EFA)

The Kaiser-Meyer-Olkin (KMO) values were 0.832 for the 16-17 age group and 0.741 for the 18-year-old group, both exceeding the recommended threshold of 0.6. Bartlett’s test of sphericity was significant for both groups (χ² (136) = 2386.343, p <.001 for 16-17; χ² (136) = 878.814, p <.001 for 18), indicating the data were suitable for factor analysis. Principal component analysis revealed that the cumulative variance explained was 62.892% for the 16-17 group and 61.649% for the 18-year-old group, suggesting good factor representation (**Supplementary Material 5**).

#### 3.3.2 Confirmatory factor analysis (CFA)

The measurement model demonstrated good fit for both age groups. For the 16-17 group: χ²/df = 1.856, SRMR = 0.0436, RMSEA = 0.042, GFI = 0.953, AGFI = 0.935, TLI = 0.949, CFI = 0.959. For the 18-year-old group: χ²/df = 1.38, SRMR = 0.0599, RMSEA = 0.044, GFI = 0.921, AGFI = 0.890, TLI = 0.933, CFI = 0.946.

Construct validity and reliability were assessed for both age groups. For the 16-17 age group, all factor loadings exceeded 0.5, composite reliabilities (C.R.) were above 0.7 (72), and the average variance extracted (AVE) ranged from 0.406 to 0.569. For the 18-year-old group, factor loadings were above 0.4, composite reliabilities exceeded 0.6, and AVE ranged from 0.376 to 0.718 (**Supplementary Table 1**). Discriminant validity was established for both age groups (**Table 3**), as the square root of the AVE for each construct was greater than its correlations with other constructs.

**Table 3:** Discriminant validity analysis: square root of AVE and correlations among latent variables for 16-17 and 18-year-old groups.

Group	Latent variables	AVE	PDC	PL	ST	NE	SWB
16-17	PDC	0.465	0.682	—	—	—	—
	PL	0.569	0.313	0.754	—	—	—
	ST	0.406	0.204	0.064	0.637	—	—
	NE	0.496	-0.273	-0.354	-0.218	0.704	—
	SWB	0.428	0.474	0.364	0.493	-0.522	0.654
18	PDC	0.417	0.646	—	—	—	—
	PL	0.718	0.309	0.847	—	—	—
	ST	0.376	0.333	0.103	0.613	—	—
	NE	0.467	-0.097	-0.133	-0.092	0.684	—
	SWB	0.410	0.274	0.001	0.534	-0.286	0.641

PDC, parental democratic communication; SWB, subjective well-being; ST, societal trust; NE, negative emotion; PL, pleasant life. The diagonal is the square root value of AVE.

### 3.4 Structural equation modeling

#### 3.4.1 Direct effect

**Figure 2:**
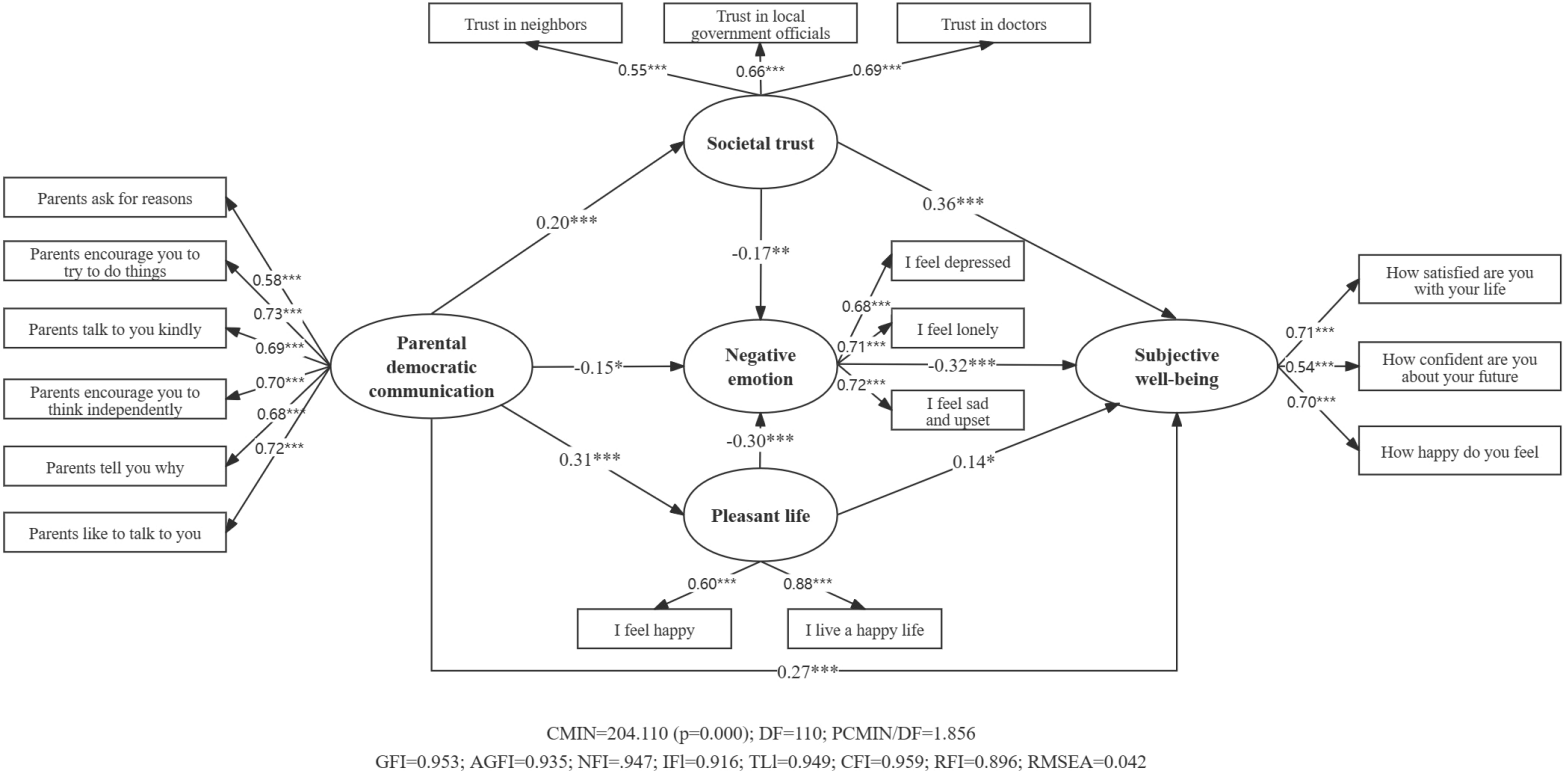
Serial mediation model of parental democratic communication’s effect on subjective well-being among 16-17-year-olds. ***p<0.001, **p<0.01, *p<0.05.

**Figure 3:**
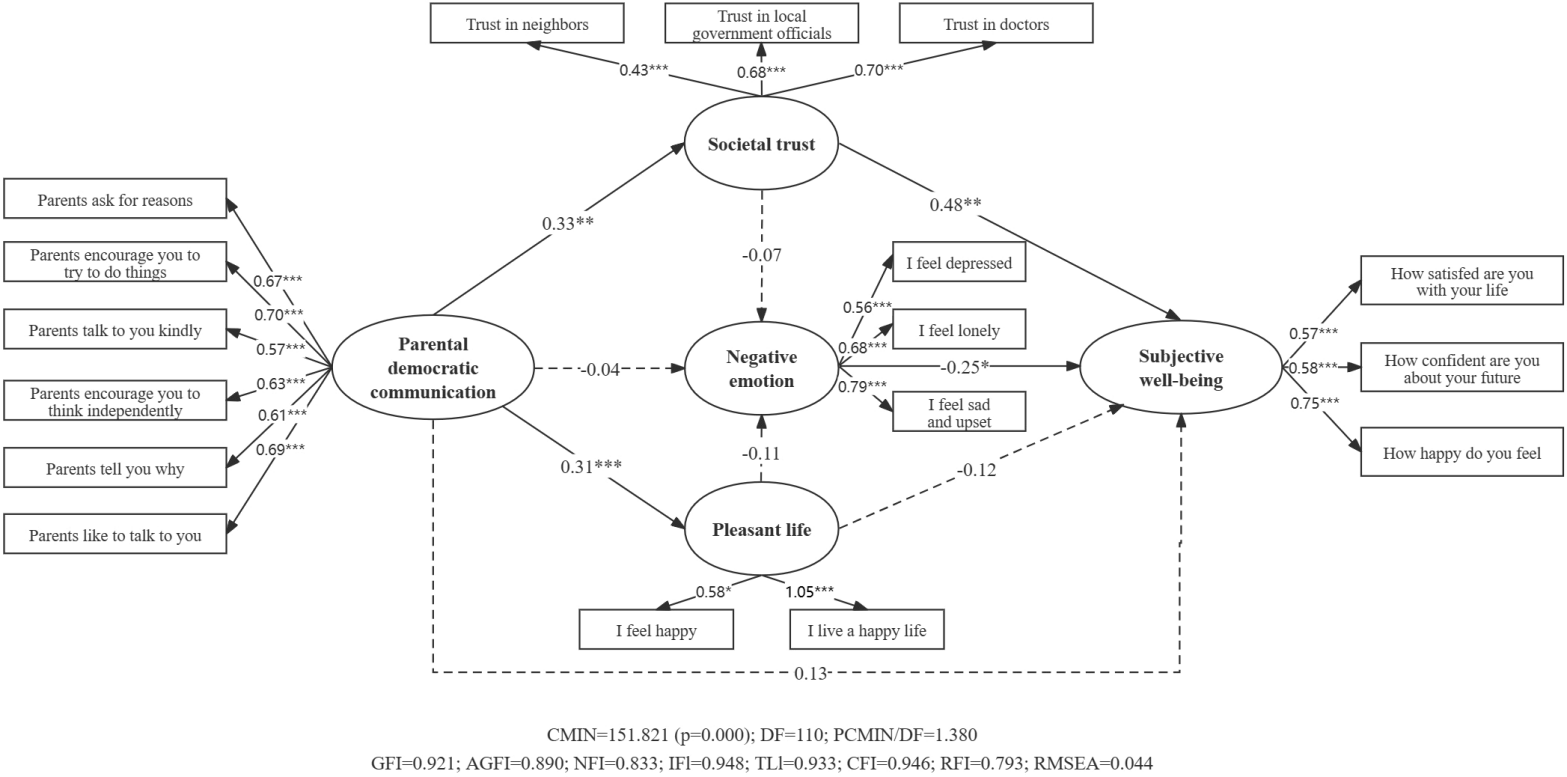
Serial mediation model of parental democratic communication’s effect on subjective well-being among 18-year-olds. ***p<0.001, **p<0.01, *p<0.05.

As shown in **Figure 2**, for the 16-17 age group, parental democratic communication significantly influenced students’ subjective well-being (β=0.269, SE=0.065, p<0.001, 95%CI [0.133, 0.39]). However, for the 18-year-old group (**Figure 3**), this effect was not significant (β=0.128, SE=0.143, p>0.05, 95% CI [-0.19, 0.375]).

#### 3.4.2 Mediation effects

In the 16-17 age group (**Figure 2**), significant mediation effects were observed for:

• Societal trust (β=0.073, SE=0.026, p<0.01, 95%CI [0.031, 0.14])

• Negative emotion (β=0.046, SE=0.027, p<0.05, 95%CI [0.005, 0.113])

• Pleasant life (β=0.045, SE=0.023, p<0.05, 95%CI [0.006, 0.097])

For the 18-year-old group (**Figure 3**), only societal trust demonstrated a significant mediation effect (β=0.16, SE=0.076, p<0.01, 95%CI [0.044, 0.368]). Negative emotion (β=0.01, SE=0.027, p>0.05, 95%CI [-0.029, 0.094]) and pleasant life (β=-0.038, SE=0.037, p>0.05, 95%CI [-0.149, 0.01]) did not show significant mediation effects.

#### 3.4.3 Serial mediation

For the 16-17 age group (**Figure 2**), the total indirect effect was significant (β=0.206, SE=0.041, p<0.01, 95%CI [0.133, 0.292]). Two significant serial mediation pathways were identified:

• Parental democratic communication →societal trust → negative emotion → well-being (β=0.011, SE=0.006, p<0.01, 95%CI [0.003, 0.027])

• Parental democratic communication → pleasant life → negative emotion → well-being (β=0.03, SE=0.011, p< 0.001, 95%CI [0.014, 0.064])

For the 18-year-old group (**Figure 3**), neither the total indirect effect (β=0.146, SE=0.086, p>0.05, 95%CI [-0.012, 0.323]) nor the two serial mediation pathways (β=0.005, SE=0.012, p>0.05, 95%CI [-0.007, 0.063]; β=0.009, SE=0.014, p>0.05, 95%CI [-0.002, 0.059]) were significant.

### 3.5 Effect size of the mediation pathways

For the 16-17 age group (**Table 4**), the total indirect effect accounted for 43.46% of the total effect, while the direct effect accounted for 56.75%. The specific effect sizes for each mediating pathway were:

**Table 4:** Mediation effects and bootstrap 95% confidence intervals for 16-17 and 18-year-old groups.

Group	Path	Estimate	SE	p	effect size	95%CI	
						Lower	Upper
16-17	PDC-ST-SWB	0.073	0.026	0.001	15.40%	0.031	0.140
	PDC-NE-SWB	0.046	0.027	0.021	9.70%	0.005	0.113
	PDC-PL-SWB	0.045	0.023	0.026	9.49%	0.006	0.097
	PDC-ST-NE-SWB	0.011	0.006	0.005	2.32%	0.003	0.027
	PDC-PL-NE-SWB	0.030	0.011	***	6.33%	0.014	0.064
	Total indirect effect	0.206	0.041	0.001	43.46%	0.133	0.292
	PDC-SWB (Direct effect)	0.269	0.065	0.001	56.75%	0.133	0.390
	Total effect	0.474	0.066	0.001	100.00%	0.336	0.593
18	PDC-ST-SWB	0.16	0.076	0.007	58.39%	0.044	0.368
	PDC-NE-SWB	0.01	0.027	0.439	—	-0.029	0.094
	PDC-PL-SWB	-0.038	0.037	0.082	—	-0.149	0.010
	PDC-ST-NE-SWB	0.005	0.012	0.244	—	-0.007	0.063
	PDC-PL-NE-SWB	0.009	0.014	0.147	—	-0.002	0.059
	Total indirect effect	0.146	0.086	0.069	—	-0.012	0.323
	PDC-SWB(Direct effect)	0.128	0.143	0.422	—	-0.190	0.375
	Total effect	0.274	0.120	0.027	100.00%	0.032	0.493

PDC, parental democratic communication; SWB, subjective well-being; ST, societal trust; NE, negative emotion; PL, pleasant life. ***p<0.001.a95%CI does not overlap with zero.

1) PDC→ST→SWB: 15.40% of total effect

2) PDC→NE→SWB: 9.70% of total effect

3) PDC→PL→SWB: 9.49% of total effect

4) PDC→ST→NE→SWB: 2.32% of total effect

5) PDC→PL→NE→SWB: 6.33% of total effect

For the 18-year-old group: only one mediating pathway showed a significant effect size: PDC→ST→SWB: 58.39% of total effect.

### 3.6 Multi-group analysis

The multi-group analysis demonstrated good overall model fit across different invariance levels. The measurement-weighted model showed stability, while other models exhibited non-significant differences. This suggests that the measurement structure is consistent across age groups, but there may be some differences in structural relationships (**Tables 5**, **6**).

**Table 5:** Model fit indices for different levels of invariance in multi-group analysis.

Model	CMIN	*df*	P	CMIN/*df*	NFI	RFI	IFI	TLI	CFI	GFI	AGFI	RMSEA	AIC	ECVI
Unconstrained	356.082	220	0	1.619	0.893	0.868	0.956	0.945	0.955	0.944	0.922	0.03	528.082	0.766
Measurement weights	372.834	232	0	1.607	0.888	0.869	0.954	0.946	0.954	0.941	0.922	0.03	520.834	0.756
Structural weights	394.375	241	0	1.636	0.881	0.866	0.95	0.943	0.95	0.939	0.922	0.03	524.375	0.761
Structural covariances	395.138	242	0	1.633	0.881	0.867	0.95	0.944	0.95	0.939	0.922	0.03	523.138	0.759
Structural residuals	396.69	246	0	1.613	0.881	0.868	0.951	0.945	0.951	0.938	0.923	0.03	516.69	0.75
Measurement residuals	437.299	263	0	1.663	0.869	0.864	0.943	0.941	0.943	0.932	0.92	0.031	523.299	0.76

**Table 6:** Changes in fit indices across invariance levels.

Model	ΔCMIN	Δ*df*	*p*	ΔNFI	ΔRFI	ΔIFI	ΔTLI	ΔCFI	ΔGFI	ΔAGFI
Measurement weights	16.752	12	0.159	-0.005	0.001	-0.002	0.001	-0.001	-0.003	0.000
Structural weights	38.293	21	0.012	-0.012	-0.002	-0.006	-0.002	-0.005	-0.005	0.000
Structural covariances	39.056	22	0.014	-0.012	-0.001	-0.006	-0.001	-0.005	-0.005	0.000
Structural residuals	40.608	26	0.034	-0.012	0.000	-0.005	0.000	-0.004	-0.006	0.001
Measurement residuals	81.217	43	0.000	-0.024	-0.004	-0.013	-0.004	-0.012	-0.012	-0.002

Path Coefficients Comparison (**Table 7**):

**Table 7:** Multi-group structural equation modeling results: path estimates for 16-17 and 18-year-old groups.

Path	Age			
	16-17		18	
	Estimate	P	Estimate	P
PDC–ST	0.204	0.001	0.333	0.006
PDC–PL	0.313	***	0.309	***
PDC–NE	-0.145	0.017	-0.04	0.708
ST–NE	-0.169	0.007	-0.067	0.551
PL–NE	-0.297	***	-0.114	0.350
ST–SWB	0.359	***	0.481	0.001
PL–SWB	0.144	0.019	-0.123	0.125
NE–SWB	-0.32	***	-0.246	0.026
PDC–SWB	0.269	***	0.128	0.218

PDC, parental democratic communication; SWB, subjective well-being; ST, societal trust; NE, negative emotion; PL, pleasant life. ***p<0.001.

1) Parental Democratic Communication (PDC) to Societal Trust (ST), both significant, slightly stronger for 18-year-olds.

2) PDC to Pleasant Life (PL), significant and similar for both groups.

3) PDC to Negative Emotion (NE), significant only for 16-17 years.

4) ST to NE, significant only for 16-17 years.

5) PL to NE, significant only for 16-17 years.

6) ST to Subjective Well-being (SWB), significant for both, stronger for 18-year-olds.

7) PL to SWB, significant only for 16-17 years, notable difference in direction. The critical ratio is 2.769 and greater than 1.96.

8) NE to SWB, significant for both, slightly stronger for 16-17 years.

9) PDC to SWB (Direct effect), significant only for 16-17 years.

## 4 Discussion

### 4.1 Summary of main findings

As highlighted in **Table 8**, our study findings suggest that the influence of parental democratic communication on subjective well-being, as well as the mediating mechanisms, differ between the two age groups. Our findings highlight the complex interplay between PDC, societal trust, negative emotions, and pleasant life experiences in shaping adolescent well-being.

**Table 8:** Summary of results: effects of parental democratic communication on adolescent well-being.

Aspect	16-17 Years Old	18 Years Old
Direct Effect (Parental Democratic Communication→ Subjective Well-being)	Significant (β = 0.269, p < 0.001)	Non-significant (β = 0.128, p > 0.05)
Total Indirect Effect	43.46% of total effect	Not significant overall
Significant Mediators	Societal trust, Negative Emotions, Pleasant Life	Societal trust only
Strongest Mediator	Societal trust (15.40% of total effect)	Societal trust (58.39% of total effect)

### 4.2 Age-related differences in the impact of parental democratic communication

Our findings reveal significant age-related differences in the impact of Parental Democratic Communication (PDC) on Subjective Well-Being (SWB) among Chinese adolescents. The direct effect of PDC on SWB was significant for mid-adolescents (16-17 years) but not for late adolescents (18 years). This shift likely reflects the changing dynamics of parent-child relationships as adolescents gain autonomy (73), prioritize independent self-construal (74), and focus more on peer relationships and identity development (75–77). These results suggest that supportive and open parent-child communication may be particularly crucial for younger adolescents (78, 79). Our findings are consistent with recent research in China that emphasizes that adolescents’ autonomy increases with age. Li (80) notes that high school students in China experience enhanced psychological resilience and autonomy as they mature, which may explain the changing dynamics in our study. Similarly, Wang and Zhang (81) emphasize the growing importance of peer relationships for Chinese adolescents, supporting our observation of diminishing direct parental influence on well-being for 18-year-olds.

However, our findings extend beyond these observations by revealing that parental influence persists, albeit through different mechanisms. The mediating role of social trust remained significant across both age groups, indicating that even as Chinese adolescents transition to young adulthood, parental communication continues to shape their perceptions of the social world. This observation challenges the notion that parental influence necessarily diminishes with increasing adolescent autonomy, a concept often emphasized in Western literature (82). Our results align with and extend previous research linking societal trust to psychological adjustment and well-being among Chinese adolescents (83, 84).

### 4.3 Mediating pathways

For mid-adolescents (16-17 years), our study revealed a complex serial mediation involving negative emotions, pleasant life experiences and societal trust. This finding aligns with existing research showing that supportive parental communication is associated with lower levels of anxiety and depression (85, 86), as well as higher levels of life satisfaction and positive affect (87, 88). Such communication appears to provide a secure foundation for building societal trust and navigating social relationships (89), while also minimizing negative emotions (46). However, our study extends these findings by demonstrating a serial mediation relationship, offering a more nuanced understanding of how parental democratic communication (PDC) influences well-being through multiple pathways simultaneously. In the Chinese context, deep family values and an emphasis on filial piety often lead parents to provide more instruction and discipline (90). Consequently, adolescents place significant weight on parental communication and advice (91–93). In addition, with the trend of delayed adulthood becoming increasingly prevalent (94), adolescents may rely more heavily on parental guidance for developing social trust, experiencing pleasant life events, and regulating emotions, all of which influence their subjective well-being.

In late adolescence, we observe a significant developmental shift where societal trust becomes the primary mediator between parental democratic communication and well-being for 18-year-olds. This transition aligns with previous research indicating that during this period, 18-year-olds diversify their social relationships, placing greater value on interactions with peers, teachers, and community members, which significantly impact their psychology and behavior (95). This shift underscores the increasing importance of social connections during the transition to adulthood, extending theories of emerging adulthood (75).

Our findings demonstrate how parental influence adapts and persists into this developmental period, particularly in the Chinese context, by identifying societal trust as a key mediator. This perspective builds upon previous studies on parenting and adolescent well-being in China, which have emphasized the enduring influence of family due to deep-rooted cultural values. These studies have highlighted that parental education and care tend to be present throughout their children’s development (96), with factors such as parental expectations and family atmosphere significantly impacting adolescents’ academic achievement and psychological well-being (97).

### 4.4 Trust-mediated well-being: a new concept

Our study reveals the paramount importance of social trust in adolescent well-being, surpassing traditionally emphasized factors such as negative emotions and pleasant life experiences. This finding led us to propose the concept of “trust-mediated well-being.” For 16-17-year-olds, societal trust significantly influences subjective well-being through both direct (β=0.359) and indirect pathways. Indirectly, societal trust reduces negative emotions (β=-0.169), which in turn affects subjective well-being (β=-0.32). The effect of pleasant life experiences (β=0.14) is considerably smaller than that of societal trust. Among 18-year-olds, the importance of societal trust increases dramatically. Its direct effect on subjective well-being (β=0.481) accounts for 58.39% of the total effect, while the impact of negative emotions decreases (β=-0.246), and pleasant life experiences cease to have a significant effect. This concept is particularly relevant in addressing the global trend of increasing loneliness and social isolation, exacerbated by “lonelygenic environments” (13), although the specific mechanisms may differ by age.

These findings challenge the traditional emphasis on emotional regulation and hedonic experiences in well-being research, suggesting a more complex and age-specific relationship between societal trust and well-being. Our results indicate that the ability to trust and feel connected to society plays a crucial role in well-being throughout adolescence, but its mechanisms appear to evolve with age. For mid-adolescents (16-17 years), our findings partially align with recent research highlighting social trust’s protective role against negative emotions, including loneliness (98–100). In this age group, societal trust not only directly enhances well-being but also shows a small but significant negative relationship with negative emotions. However, for late adolescents (18 years), while societal trust remains a significant direct contributor to well-being, its relationship with negative emotions becomes non-significant. This suggests a shift in how societal trust operates during the transition to adulthood, maintaining its importance for overall well-being but potentially becoming decoupled from immediate emotional experiences.

In the Chinese context, where rapid social changes, including the increasing prevalence of online interactions and intense academic pressure (101, 102), as well as the COVID-19 pandemic have strained traditional social bonds (103, 104), understanding the role of societal trust in well-being becomes even more critical. Our findings suggest that while societal trust consistently contributes to well-being, its relationship with negative emotions and potential loneliness may be more complex and age-dependent than previously thought.

Trust-mediated well-being aligns with emerging concepts in the third wave of positive psychology, that emphasizes the interconnectedness of individual and collective well-being (105, 106). It extends this idea by demonstrating how individual well-being is fostered through one’s connection and trust in the larger social fabric, especially evident in our findings for 18-year-olds. In the Chinese context, where collective harmony is culturally valued, our findings offer a bridge between traditional collectivist values and contemporary approaches to individual well-being. This trust-mediated well-being model provides a framework for understanding how positive family dynamics contribute to both individual and societal well-being through the cultivation of social trust.

### 4.5 Contribution to existing knowledge

Our study makes significant contributions to adolescent development and well-being research, aligning with the third wave of positive psychology:

1. “Societal Trust-Mediated Well-Being” Pattern:

We reveal a pattern where societal trust mediates the relationship between parental democratic communication and adolescent well-being, with this mediation strengthening with age. This framework bridges individual, familial, and societal levels of analysis, resonating with the holistic approach of third-wave positive psychology.

2. Extension of Emerging Adulthood Theory:

Traditionally, theories of adolescent development, including some interpretations of Arnett’s emerging adulthood theory (75), have posited that individuals become less influenced by their parents and more independent as they approach and enter adulthood around age 18. However, our study reveals a more nuanced picture. We find that the influence of parental democratic communication does not simply diminish at age 18; instead, its pathway of influence evolves. For 18-year-olds, parental influence manifests more prominently through the mechanism of societal trust, which in turn affects well-being.

3. Cultural Sensitivity:

Our research provides a nuanced understanding of how family relationships evolve and maintain their importance during this critical developmental period. This insight is particularly valuable in the Chinese context, where family ties traditionally remain strong even as young people enter adulthood, offering a culturally sensitive extension to existing theories of adolescent development and emerging adulthood.

4. Addressing Global Youth Negative Emotions including Loneliness:

In response to growing global concerns about youth loneliness and negative emotions (3, 13), our study offers a novel theoretical framework. This framework elucidates how positive family interactions can serve as a buffer against these issues through the cultivation of trust-mediated well-being. While previous research has predominantly focused on individual-level interventions to mitigate loneliness and negative emotions, our study breaks new ground by integrating family dynamics and societal trust into the equation. By examining the interplay between parental democratic communication, societal trust, and adolescent well-being, we address a significant theoretical gap in tackling these pressing social issues. Our approach provides a more comprehensive understanding of the protective factors against youth loneliness and negative emotions, emphasizing the crucial role of family relationships and broader social connections in fostering resilience and emotional well-being among adolescents and young adults.

### 4.6 Practical implications

Our findings suggest the need for integrated, age-tailored approaches in promoting adolescent well-being. For younger adolescents, interventions might focus on enhancing PDC to directly impact SWB, in addition to societal trust, emotion regulation and pleasant life experiences. For older adolescents, the focus might shift to fostering trust-mediated well-being alongside parental communication. Our results suggest the need for culturally and age-appropriate in-person interactive activities in schools and communities. Youth well-being professionals should consider incorporating societal trust-mediated well-being as part of their objectives in assessments and interventions, particularly for older adolescents.

### 4.7 Limitations and future directions

While our study utilized data from a large-scale survey, enhancing reliability and generalizability (107), several limitations should be noted:

1. Measurement Issues:

The use of secondary data limited available measures and constructs and some measures showed suboptimal internal consistency reliability. However, this can be considered acceptable due to:

a) Short scale length, which often results in lower Cronbach’s alpha values (108).

b) Construct breadth: Social trust is a broad construct involving trust in different social entities, which may lead to lower internal consistency but improve construct validity (109).

c) Age-specific considerations, with slightly lower alpha for 18-year-olds potentially reflecting developmental changes.

d) Mean inter-item correlations (ranging from.23 to.51) may be more appropriate for short scales of societal trust (109).

2. Methodological Limitations:

Reliance on self-reported measures introduces potential response bias. The cross-sectional design precludes causal inferences. Potential confounding variables (e.g., socioeconomic status, family structure) were not adequately controlled for. In addition, the data collection during the COVID-19 period may have introduced confounding effects. The interpretation of age differences should consider cohort effects and other potential confounds. Despite using data from the nationally representative CFPS, our sample selection process may have introduced some selection bias. The exclusion of respondents with missing data, particularly those who indicated parental communication was not applicable (n=142), may have systematically removed adolescents with non-traditional family structures or unique living situations. This could potentially skew our understanding of parental influence.

3. Conceptual Limitations:

Our focus on the hedonic approach to well-being may not capture all aspects of adolescent development (110).

Future research should address these limitations by:

a) Validating the paths and mechanisms in diverse ethnic groups with more comprehensive measurement scales. Employing multiple methods such as behavioral observations, physiological measures, reports from others to reduce response bias and improve data objectivity.

b) Investigating the characteristics and outcomes of adolescents for whom traditional measures of parental communication may not apply. Exploring potential boundary conditions (e.g., social-emotional competence, social media use, family structure, socioeconomic status). Utilizing parceling techniques and advanced predictive models for feature selection.

c) Conducting longitudinal studies to examine relationships between variables over time and employing experimental designs to identify causal effects.

d) Utilizing mixed-methods approaches to gain deeper insights into adolescents’ lived experiences and the nuanced influences of societal trust on well-being.

## 5 Conclusion

Our study reveals distinct age-related differences in the impact of parental communication on adolescent well-being, underscoring the critical need for age-specific interventions that foster both positive family communication and trust-mediated well-being. These findings are particularly relevant in addressing growing global concerns about youth loneliness and social disconnection, especially in rapidly evolving societies like China.

## Data Availability

Publicly available datasets were analyzed in this study. This data can be found here: https://www.isss.pku.edu.cn/cfps/.

## Footnotes

1 We conducted a principal component factor analysis, which showed that 'trust in Americans' had low correlations with other societal trust indicators and may reflect cross-cultural attitudes rather than everyday social trust (**Supplementary Material 4**), the AMOS results show that factor loading coefficient for 'trust in parents' and 'trust in strangers' were less than 0.5 (56). Consequently, these items were excluded from the final measurement.

## References

[1] Zhang R Luo Y Jiang Y Tang S. Parental emotional neglect and depression among adolescents in China: a moderated mediation model. Curr Psychol. (2024) 43:21723–34. doi: 10.1007/s12144-024-05992-9

[2] Wu L Zhang D Cheng G Hu T Rost DH. Parental emotional warmth and psychological *Suzhi* as mediators between socioeconomic status and problem behaviours in Chinese children. Child Youth Serv Rev. (2015) 59:132–8. doi: 10.1016/j.childyouth.2015.09.019

[3] Twenge JM Cooper AB Joiner TE Duffy ME Binau SG. Age, period, and cohort trends in mood disorder indicators and suicide-related outcomes in a nationally representative dataset, 2005–2017. J Abnorm Psychol. (2019) 128:185–99. doi: 10.1037/abn0000410 30869927

[4] Cosma A Stevens G Martin G Duinhof EL Walsh SD Garcia-Moya I. Cross-national time trends in adolescent mental well-being from 2002 to 2018 and the explanatory role of schoolwork pressure. J Adolesc Health. (2020) 66:S50–8. doi: 10.1016/j.jadohealth.2020.02.010 PMC8131201 32446609

[5] Rose T Joe S Williams A Harris R Betz G Stewart-Brown S. Measuring mental wellbeing among adolescents: A systematic review of instruments. J Child Fam Stud. (2017) 26:2349–62. doi: 10.1007/s10826-017-0754-0

[6] Fritz J De Graaff AM Caisley H Van Harmelen AL Wilkinson PO. A systematic review of amenable resilience factors that moderate and/or mediate the relationship between childhood adversity and mental health in young people. Front Psychiatry. (2018) 9:230. doi: 10.3389/fpsyt.2018.00230 29971021 PMC6018532

[7] Lolas F. Quality of life: objectifying the subjective experience. Alpha Psychiatr. (2023) 24:67. doi: 10.5152/alphapsychiatry.2023.280223 PMC10151985 37144049

[8] Marques De Miranda D Da Silva Athanasio B Sena Oliveira AC Simoes-e-Silva AC. How is COVID-19 pandemic impacting mental health of children and adolescents? Int J Disaster Risk Reduct. (2020) 51:101845. doi: 10.1016/j.ijdrr.2020.101845 32929399 PMC7481176

[9] Mikolajczak M Brianda ME Avalosse H Roskam I. Consequences of parental burnout: Its specific effect on child neglect and violence. Child Abuse Negl. (2018) 80:134–45. doi: 10.1016/j.chiabu.2018.03.025 29604504

[10] Guo X Hao C Wang W Li Y. Parental burnout, negative parenting style, and adolescents’ Development. Behav Sci. (2024) 14:161. doi: 10.3390/bs14030161 38540464 PMC10968024

[11] Lazer DMJ Baum MA Benkler Y Berinsky AJ Greenhill KM Menczer F. The science of fake news. Science. (2018) 359:1094–6. doi: 10.1126/science.aao2998 29590025

[12] Schröder JM Neumayr M. How socio-economic inequality affects individuals’ civic engagement: a systematic literature review of empirical findings and theoretical explanations. Socio-Econ Rev. (2023) 21:665–94. doi: 10.1093/ser/mwab058

[13] Feng X Astell-Burt T. Lonelygenic environments: a call for research on multilevel determinants of loneliness. Lancet Planet Health. (2022) 6:e933–4. doi: 10.1016/S2542-5196(22)00306-0 36495885

[14] Loades ME Chatburn E Higson-Sweeney N Reynolds S Shafran R Brigden A. Rapid systematic review: the impact of social isolation and loneliness on the mental health of children and adolescents in the context of COVID-19. J Am Acad Child Adolesc Psychiatry. (2020) 59:1218–1239.e3. doi: 10.1016/j.jaac.2020.05.009 32504808 PMC7267797

[15] Hoskins DH. Consequences of parenting on adolescent outcomes. Societies. (2014) 4:506–31. doi: 10.3390/soc4030506

[16] Samji H Wu J Ladak A Vossen C Stewart E Dove N. Review: Mental health impacts of the COVID-19 pandemic on children and youth – a systematic review. Child Adolesc Ment Health. (2022) 27:173–89. doi: 10.1111/camh.12501 PMC8653204 34455683

[17] Bi X Yang Y Li H Wang M Zhang W. Parenting styles and parent–adolescent relationships: The mediating roles of behavioral autonomy and parental authority. Front Psychol. (2018) 9:2187. doi: 10.3389/fpsyg.2018.02187 30483194 PMC6243060

[18] Fosco GM LoBraico EJ. Elaborating on premature adolescent autonomy: Linking variation in daily family processes to developmental risk. Dev Psychopathol. (2019) 31:1741–55. doi: 10.1017/S0954579419001032 PMC8719457 31455441

[19] Han CS Brussoni MJ Mâsse LC. Parental autonomy support in the context of parent–child negotiation for children’s independent mobility: ‘I always feel safer with my parents’ to ‘Boom! Bust down those walls!’. J Early Adolesc. (2022) 42:737–64. doi: 10.1177/02724316211064513 PMC9082966 35559208

[20] Ken CS Guan NC. Commentary: internalizing problems in childhood and adolescence: the role of the family. Alpha Psychiatr. (2023) 24:93–4. doi: 10.5152/alphapsychiatry.2023.080523 PMC10334622 37440898

[21] Pinquart M Gerke DC. Associations of parenting styles with self-esteem in children and adolescents: A meta-analysis. J Child Fam Stud. (2019) 28:2017–35. doi: 10.1007/s10826-019-01417-5

[22] Huang L Wu W Yang F. Parenting style and subjective well-being in children and youth: A meta-analysis. Psychol Rep. (2024), 332941241256883. doi: 10.1177/00332941241256883 38772039

[23] Wray-Lake L Flanagan CA. Parenting practices and the development of adolescents’ social trust. J Adolesc. (2012) 35:549–60. doi: 10.1016/j.adolescence.2011.09.006 PMC11378488 22015215

[24] Gao M Potwarka L. Investigating the role of family travel and family functioning in promoting Chinese adolescents’ subjective wellbeing. J Leis Res. (2021) 52:487–507. doi: 10.1080/00222216.2021.1927264

[25] Lietz P Dix KL Tarabashkina L O’Grady E Ahmed SK. Family fun: a vital ingredient of early adolescents having a good life. J Fam Stud. (2020) 26:459–76. doi: 10.1080/13229400.2017.1418410

[26] Moore GF Cox R Evans RE Hallingberg B Hawkins J Littlecott HJ. School, peer and family relationships and adolescent substance use, subjective wellbeing and mental health symptoms in wales: a cross sectional study. Child Indic Res. (2018) 11:1951–65. doi: 10.1007/s12187-017-9524-1 PMC6244918 30524519

[27] Hamama L Arazi Y. Aggressive behaviour in at-risk children: contribution of subjective well-being and family cohesion. Child & Family Social Work. (2012) 17:284–95. doi: 10.1111/j.1365-2206.2011.00779.x

[28] Gross JJ Thompson R. “Emotion Regulation: Conceptual Foundations”. In: Gross JJ, editor. Handbook of emotion regulation. The Guilford Press. (2007) pp. 3–24.

[29] Bi X Wang S. Parent-adolescent communication quality and life satisfaction: the mediating roles of autonomy and future orientation. Psychol Res Behav Manag. (2021) 14:1091–9. doi: 10.2147/PRBM.S317389 PMC8310925 34321937

[30] Zapf H Boettcher J Haukeland Y Orm S Coslar S Wiegand-Grefe S. A systematic review of parent–child communication measures: instruments and their psychometric properties. Clin Child Fam Psychol Rev. (2023) 26:121–42. doi: 10.1007/s10567-022-00414-3 PMC9879831 36166179

[31] Yang T Gai X Wang S Gai S. The relationship between parenting behaviors and adolescent well-being varies with the consistency of parent–adolescent cultural orientation. Behav Sci. (2024) 14:193. doi: 10.3390/bs14030193 38540496 PMC10967969

[32] Du W Jian M Hua F Qi S. Influence of positive parenting styles on self-regulated learning in chinese adolescents testing the mediating effects of self-esteem. Appl Res Qual Life. (2022) 17:2619–35. doi: 10.1007/s11482-021-09985-9

[33] Lim SA You S Ha D. Parental emotional support and adolescent happiness: mediating roles of self-esteem and emotional intelligence. Appl Res Qual Life. (2015) 10:631–46. doi: 10.1007/s11482-014-9344-0

[34] Ehsan A Klaas HS Bastianen A Spini D. Social capital and health: A systematic review of systematic reviews. SSM Popul Health. (2019) 8:100425. doi: 10.1016/j.ssmph.2019.100425 31431915 PMC6580321

[35] Newton K. “Social and political trust”. In: Dalton RJ Klingemann H-D, editors. The Oxford handbook of political behaviour. New York, USA: Oxford University Press. (2007). pp. 342–61. doi: 10.1093/oxfordhb/9780199270125.003.0018

[36] Kapetanovic S Rothenberg WA Lansford JE Bornstein MH Chang L Deater-Deckard K. Cross-cultural examination of links between parent–adolescent communication and adolescent psychological problems in 12 cultural groups. J Youth Adolesc. (2020) 49:1225–44. doi: 10.1007/s10964-020-01212-2 PMC7237396 32166654

[37] Cheng WY Cheung RYM Chung KKH. Understanding adolescents’ perceived social responsibility: The role of family cohesion, interdependent self-construal, and social trust. J Adolesc. (2021) 89:55–62. doi: 10.1016/j.adolescence.2021.04.001 33873101

[38] Peng B Hu N Yu H Xiao H Luo J. Parenting style and adolescent mental health: the chain mediating effects of self-esteem and psychological inflexibility. Front Psychol. (2021) 12:738170. doi: 10.3389/fpsyg.2021.738170 34721210 PMC8548717

[39] Wang J Mann F Lloyd-Evans B Ma R Johnson S. Associations between loneliness and perceived social support and outcomes of mental health problems: a systematic review. BMC Psychiatry. (2018) 18:156. doi: 10.1186/s12888-018-1736-5 29843662 PMC5975705

[40] Zhao M Li Y Lin J Fang Y Yang Y Li B. The relationship between trust and well-being: A meta-analysis. J Happiness Stud. (2024) 25:56. doi: 10.1007/s10902-024-00737-8

[41] Guo Q Zheng W Shen J Huang T Ma K. Social trust more strongly associated with well-being in individualistic societies. Pers Individ Differ. (2022) 188:111451. doi: 10.1016/j.paid.2021.111451

[42] Glatz C Eder A. Patterns of trust and subjective well-being across europe: new insights from repeated cross-sectional analyses based on the european social survey 2002–2016. Soc Indic Res. (2020) 148:417–39. doi: 10.1007/s11205-019-02212-x

[43] Steinberg L. Adolescence (12th ed. ). McGraw-Hill Educ. (2021).

[44] Dahl RE Allen NB Wilbrecht L Suleiman AB. Importance of investing in adolescence from a developmental science perspective. Nature. (2018) 554:441–50. doi: 10.1038/nature25770 29469094

[45] Yang J Zhang R Zhou K Zhou J Wang X Yang W. Exploring multiple perspectives on psychological health of adolescents in relation to gender and school grade — Jiangsu province, China, 2022. China CDC Wkly. (2024) 6:713–8. doi: 10.46234/ccdcw2024.161 PMC11264053 39050019

[46] Kemph JP Erikson EH. Identity, youth and crisis. 1968. Behav Sci. (1969) 14:154–9. doi: 10.1002/bs.3830140209

[47] Diekema DS. Adolescent brain development and medical decision-making. Pediatrics. (2020) 146:S1–S24. doi: 10.1542/peds.2020-0818f 32737228

[48] Cage E Jones E Ryan G Hughes G Spanner L. Student mental health and transitions into, through and out of university: Student and staff perspectives. J Furth High Educ. (2021) 45:1076–89. doi: 10.1080/0309877X.2021.1875203

[49] Cui L Morris AS Criss MM Houltberg BJ Silk JS. Parental psychological control and adolescent adjustment: the role of adolescent emotion regulation. Parenting. (2014) 14:47–67. doi: 10.1080/15295192.2014.880018 25057264 PMC4104177

[50] Zhang Q Pan Y Zhang L Lu H. Parent-adolescent communication and early adolescent depressive symptoms: the roles of gender and adolescents’ Age. Front Psychol. (2021) 12:647596. doi: 10.3389/fpsyg.2021.647596 34040561 PMC8141856

[51] Wu C-W Chen W-W Jen C-H. Emotional intelligence and cognitive flexibility in the relationship between parenting and subjective well-being. J Adult Dev. (2021) 28:106–15. doi: 10.1007/s10804-020-09357-x

[52] Coyne SM Padilla-Walker LM Day RD Harper J Stockdale L. A friend request from dear old dad: associations between parent-child social networking and adolescent outcomes. Cyberpsychol Behav Soc Netw. (2014) 17:8–13. doi: 10.1089/cyber.2012.0623 23845157

[53] Kong ST Wu Q. Chinese family and society dynamics using the China family panel studies (CFPS) household panel. Aust Econ Rev. (2019) 52:127–33. doi: 10.1111/1467-8462.12315

[54] Baumrind D. The influence of parenting style on adolescent competence and substance use. J Early Adolesc. (1991) 11:56–95. doi: 10.1177/0272431691111004

[55] Delhey J Newton K. Predicting cross-national levels of social trust: global pattern or Nordic exceptionalism? Eur Sociol Rev. (2005) 21:311–27. doi: 10.1093/ESR/JCI022

[56] Hair JF Black WC Babin BJ Anderson RE. Multivariate data analysis: A Global Perspective. Pearson. (2010).

[57] Raharjanti NW Wiguna T Purwadianto A Soemantri D Indriatmi W PoeRwandari EK. Translation, validity and reliability of decision style scale in forensic psychiatric setting in Indonesia. Heliyon. (2022) 8:e09810. doi: 10.1016/j.heliyon.2022.e09810 35815133 PMC9257327

[58] Huhe N Chen J Tang M. Social trust and grassroots governance in rural China. Soc Sci Res. (2015) 53:351–63. doi: 10.1016/j.ssresearch.2015.06.010 26188459

[59] Radloff LS. The CES-D scale: A self-report depression scale for research in the general population. Appl Psychol Meas. (1977) 1:385–401. doi: 10.1177/014662167700100306

[60] Wu C Wang Z. The dynamic features of emotion dysregulation in major depressive disorder: An emotion dynamics perspective. Adv Psychol Sci. (2024) 32:364–85. doi: 10.3724/SP.J.1042.2024.00364

[61] Keyes CL. Mental illness and/or mental health? Investigating axioms of the complete state model of health. J Consult Clin Psychol. (2005) 73:539–48. doi: 10.1037/0022-006X.73.3.539 15982151

[62] Wang X Zhang D Wang J. Dual-factor model of mental health: Surpass the traditional mental health model. Psychol. (2011) 2:767–72. doi: 10.4236/psych.2011.28117

[63] Kline P. An easy guide to factor analysis (1st ed.). London, United Kingdom: Routledge (1994). doi: 10.4324/9781315788135

[64] Wen Z Ye B. Analyses of mediating effects: the development of methods and models. Adv Psychol Sci. (2014) 22:731. doi: 10.3724/SP.J.1042.2014.00731

[65] Anderson JC Gerbing DW. Structural equation modeling in practice: A review and recommended two-step approach. Psychol Bull. (1988) 103:411–23. doi: 10.1037/0033-2909.103.3.411

[66] Preacher KJ Hayes AF. Asymptotic and resampling strategies for assessing and comparing indirect effects in multiple mediator models. Behav Res Methods. (2008) 40:879–91. doi: 10.3758/BRM.40.3.879 18697684

[67] Byrne BM. Structural equation modeling with AMOS: basic concepts, applications, and programming. 3rd ed. New York: Routledge (2016). doi: 10.4324/9781315757421

[68] Wu ML. Structural equation modeling - AMOS operations and applications. Chongqing: Chongqing Univ Press. (2010).

[69] Saltelli A Chan K Scott EM. Sensitivity analysis. Wiley. (2009).

[70] Hao Z Lirong L. Statistical remedies for common method biases. Adv Psychol Sci. (2004) 12:942. Available online at: https://journal.psych.ac.cn/adps/EN/abstract/abstract894.shtml.

[71] Kline RB. Principles and practice of structural equation modeling. 4th ed. New York, NY, US: Guilford Press (2016). p. 534.

[72] Zhang Z Zheng L. Consumer community cognition, brand loyalty, and behaviour intentions within online publishing communities: An empirical study of Epubit in China. Learn Publ. (2021) 34:116–27. doi: 10.1002/leap.1327

[73] Smetana JG Campione-Barr N Metzger A. Adolescent development in interpersonal and societal contexts. Annu Rev Psychol. (2006) 57:255–84. doi: 10.1146/annurev.psych.57.102904.190124 16318596

[74] Cheng C Jose PE Sheldon KM Singelis TM Cheung MWL Tiliouine H. Sociocultural differences in self-construal and subjective well-being: A test of four cultural models. J Cross-Cult Psychol. (2011) 42:832–55. doi: 10.1177/0022022110381117

[75] Arnett JJ. Emerging adulthood: A theory of development from the late teens through the twenties. Am Psychol. (2000) 55:469–80. doi: 10.1037/0003-066X.55.5.469 10842426

[76] Metzger A Ice C Cottrell L. But I trust my teen: parents′ Attitudes and response to a parental monitoring intervention. AIDS Res Treat. (2012) 2012:1–10. doi: 10.1155/2012/396163 PMC3376478 22720144

[77] Villalobos Solís M Smetana JG Comer J. Associations among solicitation, relationship quality, and adolescents’ disclosure and secrecy with mothers and best friends. J Adolesc. (2015) 43:193–205. doi: 10.1016/j.adolescence.2015.05.016 26142840

[78] Ackard DM Neumark-Sztainer D Story M Perry C. Parent–child connectedness and behavioral and emotional health among adolescents. Am J Prev Med. (2006) 30:59–66. doi: 10.1016/j.amepre.2005.09.013 16414425

[79] Jackson S Bijstra J Oostra L Bosma H. Adolescents’ perceptions of communication with parents relative to specific aspects of relationships with parents and personal development. J Adolesc. (1998) 21:305–22. doi: 10.1006/jado.1998.0155 9657897

[80] Li J. Factors influencing psychological resilience of high school students and methods for improvement [In Chinese]. Tianjin Educ. (2024) 25:19–20.

[81] Wang Y Zhang Q. Collaborative education: reflections and practices on constructing a “Research interchange” for regional moral education. Pudong Educ. (2024) 7:20–4.

[82] Steinberg L Silverberg SB. The vicissitudes of autonomy in early adolescence. Child Dev. (1986) 57:841–51. doi: 10.2307/1130361 3757604

[83] Lau M Li W. The extent of family and school social capital promoting positive subjective well-being among primary school children in Shenzhen, China. Child Youth Serv Rev. (2011) 33:1573–82. doi: 10.1016/j.childyouth.2011.03.024

[84] Eagle DE Hybels CF Proeschold-Bell RJ. Perceived social support, received social support, and depression among clergy. J Soc Pers Relatsh. (2019) 36:2055–73. doi: 10.1177/0265407518776134

[85] Ma C Ma Y Wang Y. Parental autonomy support and mental health among chinese adolescents and emerging adults: the mediating role of self-esteem. Int J Environ Res Public Health. (2022) 19:14029. doi: 10.3390/ijerph192114029 36360911 PMC9653793

[86] Wang M-T Henry DA Smith LV Huguley JP Guo J. Parental ethnic-racial socialization practices and children of color’s psychosocial and behavioral adjustment: A systematic review and meta-analysis. Am Psychol. (2020) 75:1–22. doi: 10.1037/amp0000464 31058521

[87] Cava M-J Buelga S Musitu G. Parental communication and life satisfaction in adolescence. Span J Psychol. (2014) 17:E98. doi: 10.1017/sjp.2014.107 26055552

[88] Levin KA Dallago L Currie C. The association between adolescent life satisfaction, family structure, family affluence and gender differences in parent–child communication. Soc Indic Res. (2012) 106:287–305. doi: 10.1007/s11205-011-9804-y

[89] Gurdal S Lansford JE Sorbring E. Parental perceptions of children’s agency: Parental warmth, school achievement and adjustment. Early Child Dev Care. (2015) 186:1203–11. doi: 10.1080/03004430.2015.1083559 PMC4999070 27570362

[90] Hsu FLK. Americans and chinese: passages to differences. Univ Hawaii Press. (1981). doi: 10.1515/9780824845124

[91] Chen X Liu M Li D. Parental warmth, control, and indulgence and their relations to adjustment in Chinese children: A longitudinal study. J Fam Psychol. (2000) 14:401–19. doi: 10.1037/0893-3200.14.3.401 11025932

[92] Shek DTL Sun RCF. Parenting in hong kong: traditional chinese cultural roots and contemporary phenomena. In: Selin H, editor. Parenting across cultures: childrearing, motherhood and fatherhood in non-western cultures. Springer Netherlands, Dordrecht (2014). p. 25–38. doi: 10.1007/978-94-007-7503-9_3

[93] Hiniker A Schoenebeck SY Kientz JA. “Not at the dinner tabl: parents’ and children’s perspectives on family technology rules”, in: Proceedings of the 19th ACM Conference on Computer-Supported Cooperative Work & Social Computing (CSCW '16). New York, NY, USA: Association for Computing Machinery. (2016), 1376–89. doi: 10.1145/2818048.2819940

[94] Ebert C. Laurence steinberg: age of opportunity: lessons from the new science of adolescence. J Youth Adolesc. (2015) 44:1652–5. doi: 10.1007/s10964-015-0277-1

[95] Jiang X. A study on the localization of the youth quality of life scale (YQOL-R). (Doctoral dissertation, Master's thesis). Zhejiang University, China (In Chinese) (2014).

[96] Yu G Zhang Z. A review of the impact of household noise on the mental health of children and adolescents. J Shanxi Norm Univ (Soc Sci Ed). (2024) 2024:1–8. doi: 10.16207/j.cnki.1001-5957.20240919.001

[97] Tang T Wang Y Gong F Shi K Li X Liu W. Parenting styles and positive development of chinese adolescents: A meta-analysis. Adv Psychol Sci. (2024) 32:1302–19. doi: 10.3724/SP.J.1042.2024.01302

[98] Goodfellow C Hardoon D Inchley J Leyland AH Qualter P Simpson SA. Loneliness and personal well-being in young people: Moderating effects of individual, interpersonal, and community factors. J Adolesc. (2022) 94:554–68. doi: 10.1002/jad.12046 PMC9320932 35403218

[99] Akgül H Güven AZ Güven S Ceylan M. Loneliness, social support, social trust, and subjective wellness in low-income children: A longitudinal approach. Children. (2023) 10:1433. doi: 10.3390/children10091433 37761396 PMC10529055

[100] Hou T Xie Y Mao X Liu Y Zhang J Wen J. The mediating role of loneliness between social support and depressive symptoms among chinese rural adolescents during COVID-19 outbreak: A comparative study between left-behind and non-left-behind students. Front Psychiatry. (2021) 12:740094. doi: 10.3389/fpsyt.2021.740094 34497549 PMC8420998

[101] Jiang Z Chu X Yuan W Song Y Lin Z Liu Y. The role of peer support in promoting mental health of chinese adolescents. China CDC Wkly. (2024) 6:723–6. doi: 10.46234/ccdcw2024.163 PMC11264051 39050016

[102] Deng Y Cherian J Khan NUN Kumari K Sial MS Comite U. Family and academic stress and their impact on students’ Depression level and academic performance. Front Psychiatry. (2022) 13:869337. doi: 10.3389/fpsyt.2022.869337 35782431 PMC9243415

[103] Valkenburg PM Meier A Beyens I. Social media use and its impact on adolescent mental health: An umbrella review of the evidence. Curr Opin Psychol. (2022) 44:58–68. doi: 10.1016/j.copsyc.2021.08.017 34563980

[104] Cho H Li P Ngien A Tan MG Chen A Nekmat E. The bright and dark sides of social media use during COVID-19 lockdown: Contrasting social media effects through social liability vs. social support. Comput Hum Behav. (2023) 146:107795. doi: 10.1016/j.chb.2023.107795 PMC10123536 37124630

[105] Lomas T Waters L Williams P Oades LG Kern ML. Third wave positive psychology: broadening towards complexity. J Posit Psychol. (2021) 16:660–74. doi: 10.1080/17439760.2020.1805501

[106] Wissing MP. Beyond the “Third wave of positive psychology”: challenges and opportunities for future research. Front Psychol. (2022) 12:795067. doi: 10.3389/fpsyg.2021.795067 35095679 PMC8795509

[107] Yang Y Fan S Chen W Wu Y. Broader open data needed in psychiatry: practice from the psychology and behavior investigation of Chinese residents. Alpha Psychiatr. (2024) 25:564–5. doi: 10.5152/alphapsychiatry.2024.241804 PMC11443289 39360297

[108] Cortina JM. What is coefficient alpha? An examination of theory and applications. J Appl Psychol. (1993) 78:98–104. doi: 10.1037/0021-9010.78.1.98

[109] Briggs SR Cheek JM. The role of factor analysis in the development and evaluation of personality scales. J Pers. (1986) 54:106–48. doi: 10.1111/j.1467-6494.1986.tb00391.x

[110] Ryan RM Deci EL. On happiness and human potentials: A review of research on hedonic and eudaimonic well-being. Annu Rev Psychol. (2001) 52:141–66. doi: 10.1146/annurev.psych.52.1.141 11148302

