# Supplementary material for: Parental democratic communication and adolescent well-being in an era of loneliness: the mediating role of societal trust
Source: Front Psychiatry. 2024 Nov 28;15:1500937. doi: 10.3389/fpsyt.2024.1500937 (PMC11635610; doi:10.3389/fpsyt.2024.1500937)
Supplement: Supplementary file 1 [file Table1.docx]

Supplementary material

Parental Communication and Adolescent Well-Being in an Era of Loneliness: The Mediating Role of Societal Trust

Table of contents

[Appendix panels 2](#_Toc180223648)

[**Supplementary Table 1** Latent construal reliability analysis 2](#_Toc180223649)

[**Supplementary material 2** Sensitivity analysis 5](#_Toc180223650)

[**Supplementary material 3** Power Analysis-semPower package 2.1.1 of R version 4.3.1 (2023-06-16 ucrt) 6](#_Toc180223651)

[**Supplementary material 4** Original -EFA 7](#_Toc180223652)

[**Supplementary material 5** After the present study -EFA 15](#_Toc180223653)

# Appendix panels

## **Supplementary Table 1** Latent construal reliability analysis

| Group | Latent variables | Observed variables | Std. | Unstd. | S.E. | t-value | P | SMC | C.R. | AVE |
| --- | --- | --- | --- | --- | --- | --- | --- | --- | --- | --- |
| 16-17 | PDC | qm206_b_2 | 0.717 | 1 | — | — | — | 0.514 | 0.839 | 0.465 |
|  |  | qm205_b_2 | 0.679 | 1.058 | 0.078 | 13.571 | *** | 0.461 |  |  |
|  |  | qm204_b_2 | 0.696 | 1.084 | 0.079 | 13.702 | *** | 0.484 |  |  |
|  |  | qm203_b_2 | 0.687 | 0.978 | 0.071 | 13.704 | *** | 0.472 |  |  |
|  |  | qm202_b_2 | 0.728 | 1.048 | 0.072 | 14.457 | *** | 0.530 |  |  |
|  |  | qm201_b_2 | 0.575 | 0.916 | 0.080 | 11.478 | *** | 0.331 |  |  |
|  | ST | qn10022 | 0.555 | 1 | — | — | — | 0.308 | 0.670 | 0.406 |
|  |  | qn10025 | 0.661 | 1.312 | 0.156 | 8.401 | *** | 0.437 |  |  |
|  |  | qn10026 | 0.687 | 1.124 | 0.142 | 7.891 | *** | 0.472 |  |  |
|  | PL | qn416 | 0.880 | 1 | — | — | — | 0.774 | 0.718 | 0.569 |
|  |  | qn412 | 0.603 | 0.747 | 0.117 | 6.364 | *** | 0.364 |  |  |
|  | SWB | qn12012 | 0.711 | 1 | — | — | — | 0.506 | 0.688 | 0.428 |
|  |  | qn12016 | 0.541 | 0.804 | 0.078 | 10.254 | *** | 0.293 |  |  |
|  |  | qm2016 | 0.696 | 0.982 | 0.094 | 10.408 | *** | 0.484 |  |  |
|  | NE | qn418 | 0.722 | 1 | — | — | — | 0.521 | 0.746 | 0.496 |
|  |  | qn414 | 0.712 | 0.955 | 0.081 | 11.853 | *** | 0.507 |  |  |
|  |  | qn406 | 0.677 | 1.058 | 0.090 | 11.787 | *** | 0.458 |  |  |
| 18 | PDC | qm206_b_2 | 0.687 | 1 | — | — | — | 0.472 | 0.810 | 0.417 |
|  |  | qm205_b_2 | 0.607 | 0.889 | 0.119 | 7.468 | *** | 0.368 |  |  |
|  |  | qm204_b_2 | 0.634 | 0.928 | 0.128 | 7.244 | *** | 0.402 |  |  |
|  |  | qm203_b_2 | 0.567 | 0.621 | 0.092 | 6.775 | *** | 0.321 |  |  |
|  |  | qm202_b_2 | 0.701 | 1.015 | 0.131 | 7.777 | *** | 0.491 |  |  |
|  |  | qm201_b_2 | 0.667 | 0.999 | 0.126 | 7.922 | *** | 0.445 |  |  |
|  | ST | qn10022 | 0.428 | 1 | — | — | — | 0.183 | 0.635 | 0.376 |
|  |  | qn10025 | 0.676 | 1.746 | 0.373 | 4.678 | *** | 0.457 |  |  |
|  |  | qn10026 | 0.699 | 1.522 | 0.378 | 4.027 | *** | 0.489 |  |  |
|  | NE | qn418 | 0.790 | 1 | — | — | — | 0.624 | 0.721 | 0.467 |
|  |  | qn414 | 0.679 | 0.853 | 0.140 | 6.099 | *** | 0.461 |  |  |
|  |  | qn406 | 0.563 | 0.861 | 0.147 | 5.857 | *** | 0.317 |  |  |
|  | PL | qn416 | 1.046 | 1 | — | — | — | 1.094 | 0.825 | 0.718 |
|  |  | qn412 | 0.585 | 0.59 | 0.238 | 2.482 | 0.013 | 0.342 |  |  |
|  | SWB | qn12012 | 0.573 | 1 | — | — | — | 0.328 | 0.673 | 0.410 |
|  |  | qn12016 | 0.581 | 0.884 | 0.158 | 5.585 | *** | 0.338 |  |  |
|  |  | qm2016 | 0.752 | 1.114 | 0.208 | 5.350 | *** | 0.566 |  |  |
| *Notes:* PDC=parental democratic communication; SWB=subjective well-being; ST=societal trust; NE=negative emotion; PL=pleasant life. ***p＜0.001.a95%CI does not overlap with zero. | | | | | | | | | | |

## **Supplementary material 2** Sensitivity analysis

| Different models | Group | CFI | TLI | RMSEA | SRMR |
| --- | --- | --- | --- | --- | --- |
| Original  ML, convergence from 1E-05 to 0.001, iteration 50, bootstrap 2000 | 16-17 | 0.959 | 0.949 | 0.042 | 0.0436 |
|  | 18 | 0.946 | 0.933 | 0.044 | 0.0599 |
| ML, convergence from 1E-06 to 0.001, iteration 100, bootstrap 2000 | 16-17 | 0.959 | 0.949 | 0.042 | 0.0436 |
|  | 18 | 0.946 | 0.933 | 0.044 | 0.0599 |
| ML, convergence from 1E-05 to 0.001, iteration 50, bootstrap 5000 | 16-17 | 0.959 | 0.949 | 0.042 | 0.0436 |
|  | 18 | 0.946 | 0.933 | 0.044 | 0.0599 |
| ML, convergence from 1E-06 to 0.001, iteration 100, bootstrap 5000 | 16-17 | 0.959 | 0.949 | 0.042 | 0.0436 |
|  | 18 | 0.946 | 0.933 | 0.044 | 0.0599 |
| GLS, convergence from 1E-05 to 0.001, iteration 50, bootstrap 2000 | 16-17 | 0.873 | 0.843 | 0.037 | 0.0453 |
|  | 18 | 0.858 | 0.825 | 0.037 | 0.0599 |
| GLS, convergence from 1E-06 to 0.001, iteration 100, bootstrap 5000 | 16-17 | 0.873 | 0.843 | 0.037 | 0.0453 |
|  | 18 | 0.858 | 0.825 | 0.037 | 0.0599 |
| Alternative Models - PL and NE Position Swap (NE→PL)  ML, convergence from 1E-05 to 0.001, iteration 50, bootstrap 2000 | 16-17 | 0.956 | 0.946 | 0.043 | 0.0477 |
|  | 18 | 0.947 | 0.934 | 0.044 | 0.0583 |
| Notes: Invariance tests for multi-groups have been tested in the article. | | | | | |

## **Supplementary material 3** Power Analysis-semPower package 2.1.1 of R version 4.3.1 (2023-06-16 ucrt)

library(semPower)

powerAnalysis <- semPower.aPriori(effect = 0.044 (18)/ 0.042 (16-17),

effect.measure = 'RMSEA',

alpha = 0.05,

power = 0.80,

df = 110)

print(powerAnalysis)

## **Supplementary material 4** Original -EFA

KMO

| KMO | | 16-17 | 18 |
| --- | --- | --- | --- |
| KMO Quantity of Sample Suitability | | 0.841 | 0.751 |
| Bartlett's test of sphericity | Approximate chi-square (math.) | 3335.789 | 1289.636 |
|  | Degrees of freedom | 253 | 253 |
|  | P | 0 | 0 |

Common Factor Variance

| Features | 16-17 | | 18 |
| --- | --- | --- | --- |
|  | Initial | Extraction | Extraction |
| Parents will ask why | 1 | 0.441 | 0.59 |
| Parents encourage you to make an effort | 1 | 0.608 | 0.566 |
| Parents talk to you kindly | 1 | 0.576 | 0.552 |
| Parents encourage you to think independently | 1 | 0.59 | 0.522 |
| Parents will tell you why | 1 | 0.57 | 0.539 |
| Parents like to talk to you | 1 | 0.577 | 0.641 |
| Trust in neighbors | 1 | 0.563 | 0.571 |
| Trust in local government officials | 1 | 0.512 | 0.593 |
| Trust in doctors | 1 | 0.539 | 0.51 |
| Trust in parents | 1 | 0.342 | 0.581 |
| Trust in Americans | 1 | 0.689 | 0.756 |
| Trust in strangers | 1 | 0.704 | 0.678 |
| I feel emotionally drained | 1 | 0.546 | 0.639 |
| I feel overwhelmed by everything I do | 1 | 0.551 | 0.557 |
| I don't sleep well | 1 | 0.342 | 0.311 |
| I feel lonely | 1 | 0.569 | 0.599 |
| I feel sad and upset | 1 | 0.592 | 0.578 |
| I feel unable to get on with my life | 1 | 0.383 | 0.438 |
| I feel happy | 1 | 0.753 | 0.774 |
| I am happy with my life | 1 | 0.738 | 0.822 |
| Satisfaction with my life | 1 | 0.549 | 0.564 |
| Level of confidence in my future | 1 | 0.456 | 0.549 |
| How happy | 1 | 0.538 | 0.668 |
| Notes: Extraction method: principal component analysis. | | | |

Total Variance Explained

| Age | Element | Initial eigenvalue | | | Extracted Load Sum of Squares | | | Rotated Load Sum of Squares | | |
| --- | --- | --- | --- | --- | --- | --- | --- | --- | --- | --- |
|  |  | Total | Variance % | Cumulative % | Total | Variance % | Cumulative % | Total | Variance % | Cumulative % |
| 16-17 | 1 | 5.255 | 22.846 | 22.846 | 5.255 | 22.846 | 22.846 | 3.459 | 15.038 | 15.038 |
|  | 2 | 2.527 | 10.986 | 33.832 | 2.527 | 10.986 | 33.832 | 3.201 | 13.92 | 28.958 |
|  | 3 | 2.07 | 9.002 | 42.834 | 2.07 | 9.002 | 42.834 | 2.48 | 10.784 | 39.742 |
|  | 4 | 1.535 | 6.675 | 49.509 | 1.535 | 6.675 | 49.509 | 2.008 | 8.732 | 48.474 |
|  | 5 | 1.336 | 5.809 | 55.318 | 1.336 | 5.809 | 55.318 | 1.574 | 6.844 | 55.318 |
|  | 6 | 0.978 | 4.253 | 59.571 |  |  |  |  |  |  |
|  | 7 | 0.909 | 3.953 | 63.525 |  |  |  |  |  |  |
|  | 8 | 0.769 | 3.343 | 66.868 |  |  |  |  |  |  |
|  | 9 | 0.741 | 3.221 | 70.089 |  |  |  |  |  |  |
|  | 10 | 0.706 | 3.07 | 73.158 |  |  |  |  |  |  |
|  | 11 | 0.631 | 2.743 | 75.902 |  |  |  |  |  |  |
|  | 12 | 0.598 | 2.601 | 78.503 |  |  |  |  |  |  |
|  | 13 | 0.548 | 2.383 | 80.885 |  |  |  |  |  |  |
|  | 14 | 0.542 | 2.357 | 83.242 |  |  |  |  |  |  |
|  | 15 | 0.527 | 2.293 | 85.535 |  |  |  |  |  |  |
|  | 16 | 0.482 | 2.096 | 87.631 |  |  |  |  |  |  |
|  | 17 | 0.477 | 2.074 | 89.705 |  |  |  |  |  |  |
|  | 18 | 0.453 | 1.971 | 91.675 |  |  |  |  |  |  |
|  | 19 | 0.426 | 1.854 | 93.529 |  |  |  |  |  |  |
|  | 20 | 0.4 | 1.741 | 95.269 |  |  |  |  |  |  |
|  | 21 | 0.382 | 1.661 | 96.93 |  |  |  |  |  |  |
|  | 22 | 0.366 | 1.59 | 98.52 |  |  |  |  |  |  |
|  | 23 | 0.34 | 1.48 | 100 |  |  |  |  |  |  |
| 18 | 1 | 4.345 | 18.889 | 18.889 | 4.345 | 18.889 | 18.889 | 3.127 | 13.594 | 13.594 |
|  | 2 | 2.863 | 12.447 | 31.336 | 2.863 | 12.447 | 31.336 | 2.924 | 12.713 | 26.308 |
|  | 3 | 2.033 | 8.838 | 40.174 | 2.033 | 8.838 | 40.174 | 2.178 | 9.47 | 35.778 |
|  | 4 | 1.643 | 7.145 | 47.319 | 1.643 | 7.145 | 47.319 | 1.967 | 8.552 | 44.33 |
|  | 5 | 1.462 | 6.357 | 53.676 | 1.462 | 6.357 | 53.676 | 1.735 | 7.544 | 51.874 |
|  | 6 | 1.251 | 5.438 | 59.114 | 1.251 | 5.438 | 59.114 | 1.665 | 7.24 | 59.114 |
|  | 7 | 0.95 | 4.13 | 63.244 |  |  |  |  |  |  |
|  | 8 | 0.883 | 3.839 | 67.083 |  |  |  |  |  |  |
|  | 9 | 0.796 | 3.459 | 70.542 |  |  |  |  |  |  |
|  | 10 | 0.753 | 3.275 | 73.818 |  |  |  |  |  |  |
|  | 11 | 0.677 | 2.943 | 76.76 |  |  |  |  |  |  |
|  | 12 | 0.651 | 2.83 | 79.59 |  |  |  |  |  |  |
|  | 13 | 0.559 | 2.43 | 82.02 |  |  |  |  |  |  |
|  | 14 | 0.529 | 2.301 | 84.321 |  |  |  |  |  |  |
|  | 15 | 0.516 | 2.244 | 86.564 |  |  |  |  |  |  |
|  | 16 | 0.49 | 2.129 | 88.693 |  |  |  |  |  |  |
|  | 17 | 0.468 | 2.034 | 90.727 |  |  |  |  |  |  |
|  | 18 | 0.458 | 1.993 | 92.72 |  |  |  |  |  |  |
|  | 19 | 0.409 | 1.78 | 94.501 |  |  |  |  |  |  |
|  | 20 | 0.355 | 1.543 | 96.044 |  |  |  |  |  |  |
|  | 21 | 0.333 | 1.447 | 97.491 |  |  |  |  |  |  |
|  | 22 | 0.325 | 1.413 | 98.904 |  |  |  |  |  |  |
|  | 23 | 0.252 | 1.096 | 100 |  |  |  |  |  |  |
| Notes: Extraction method: principal component analysis. | | | | | | | | | | |

The rotated component matrix a

| Features | 16-17 Element | | | | | 18 Element | | | | | |
| --- | --- | --- | --- | --- | --- | --- | --- | --- | --- | --- | --- |
|  | 1 | 2 | 3 | 4 | 5 | 1 | 2 | 3 | 4 | 5 | 6 |
| Parents will ask why | 0.629 | -0.122 | 0.152 |  |  | 0.76 |  |  |  |  |  |
| Parents encourage you to make an effort | 0.76 | -0.106 | 0.109 |  |  | 0.703 |  | 0.142 |  |  | 0.199 |
| Parents talk to you kindly | 0.73 |  |  | 0.116 | 0.14 | 0.597 |  | 0.411 |  |  | -0.106 |
| Parents encourage you to think independently | 0.755 |  |  |  | 0.125 | 0.634 |  | 0.211 | 0.23 | -0.107 | 0.106 |
| Parents will tell you why | 0.749 |  |  |  |  | 0.726 |  |  |  |  |  |
| Parents like to talk to you | 0.737 |  | 0.138 |  |  | 0.753 |  |  |  | 0.198 | 0.175 |
| Trust in neighbors | | -0.138 | 0.525 | 0.512 |  | 0.114 |  | 0.558 |  | 0.488 |  |
| Trust in local government officials | |  | 0.532 | 0.427 | 0.205 |  |  | 0.722 | 0.11 | 0.204 | 0.106 |
| Trust in doctors | |  | 0.685 | 0.254 |  |  |  | 0.61 | 0.353 |  |  |
| Trust in parents | 0.258 | -0.125 | 0.507 |  |  | 0.317 |  | 0.683 |  | -0.101 |  |
| Trust in Americans | | -0.109 |  | 0.819 |  |  |  |  | 0.114 | 0.853 |  |
| Trust in strangers | |  |  | 0.835 |  |  |  | 0.252 |  | 0.777 |  |
| I feel emotionally drained | | 0.725 | -0.12 |  |  |  | 0.743 | -0.264 | 0.103 |  |  |
| I feel overwhelmed by everything I do | | 0.725 | -0.135 |  |  |  | 0.743 |  |  |  |  |
| I don't sleep well |  | 0.566 | -0.116 |  |  |  | 0.53 |  | -0.152 |  |  |
| I feel lonely | -0.101 | 0.725 | -0.102 |  | -0.145 |  | 0.734 | 0.132 | -0.155 | -0.119 |  |
| I feel sad and upset | | 0.752 |  |  |  |  | 0.723 |  | -0.186 |  |  |
| I feel unable to get on with my life | -0.181 | 0.577 |  | -0.129 |  |  | 0.618 |  |  |  | -0.217 |
| I feel happy |  |  |  |  | 0.854 |  | -0.114 |  | 0.161 |  | 0.853 |
| I am happy with my life | 0.166 | -0.227 |  |  | 0.808 | 0.21 |  | 0.107 |  |  | 0.869 |
| Satisfaction with my life | 0.195 | -0.354 | 0.594 | -0.18 |  | 0.14 | -0.174 |  | 0.716 |  |  |
| Level of confidence in my future | | -0.292 | 0.574 | -0.192 |  |  | -0.125 | 0.117 | 0.717 |  |  |
| How happy | 0.274 | -0.184 | 0.608 |  | 0.243 |  |  | 0.261 | 0.767 |  |  |
| Notes: Extraction method: principal component analysis. Rotation method: Kaiser normalized maximum variance method. a Rotation has converged after 6 iterations. | | | | | | | | | | | |

## **Supplementary material 5** After the present study -EFA

KMO

| KMO | | 16-17 | 18 |
| --- | --- | --- | --- |
| KMO Quantity of Sample Suitability | | 0.832 | 0.741 |
| Bartlett's test of sphericity | Approximate chi-square (math.) | 2386.343 | 878.814 |
|  | Degrees of freedom | 136 | 136 |
|  | P | 0 | 0 |

Common Factor Variance

| Features | 16-17 | | 18 |
| --- | --- | --- | --- |
|  | Initial | Extraction | Extraction |
| Parents will ask why | 1 | 0.443 | 0.574 |
| Parents encourage you to make an effort | 1 | 0.613 | 0.574 |
| Parents talk to you kindly | 1 | 0.608 | 0.505 |
| Parents encourage you to think independently | 1 | 0.593 | 0.528 |
| Parents will tell you why | 1 | 0.565 | 0.521 |
| Parents like to talk to you | 1 | 0.584 | 0.594 |
| Trust in neighbors | 1 | 0.503 | 0.433 |
| Trust in local government officials | 1 | 0.698 | 0.702 |
| Trust in doctors | 1 | 0.64 | 0.584 |
| I feel emotionally drained | 1 | 0.649 | 0.641 |
| I feel lonely | 1 | 0.648 | 0.652 |
| I feel sad and upset | 1 | 0.688 | 0.712 |
| I feel happy | 1 | 0.755 | 0.796 |
| I am happy with my life | 1 | 0.752 | 0.822 |
| Satisfaction with my life | 1 | 0.701 | 0.604 |
| Level of confidence in my future | 1 | 0.68 | 0.578 |
| How happy | 1 | 0.57 | 0.662 |
| Notes: Extraction method: principal component analysis. | | | |

Total Variance Explained

| Age | Element | Initial eigenvalue | | | Extracted Load Sum of Squares | | | Rotated Load Sum of Squares | | |
| --- | --- | --- | --- | --- | --- | --- | --- | --- | --- | --- |
|  |  | Total | Variance % | Cumulative % | Total | Variance % | Cumulative % | Total | Variance % | Cumulative % |
| 16-17 | 1 | 4.555 | 26.794 | 26.794 | 4.555 | 26.794 | 26.794 | 3.393 | 19.958 | 19.958 |
|  | 2 | 2.181 | 12.831 | 39.625 | 2.181 | 12.831 | 39.625 | 2.055 | 12.087 | 32.045 |
|  | 3 | 1.586 | 9.33 | 48.955 | 1.586 | 9.33 | 48.955 | 1.892 | 11.128 | 43.173 |
|  | 4 | 1.295 | 7.615 | 56.57 | 1.295 | 7.615 | 56.57 | 1.757 | 10.335 | 53.509 |
|  | 5 | 1.075 | 6.322 | 62.892 | 1.075 | 6.322 | 62.892 | 1.595 | 9.384 | 62.892 |
|  | 6 | 0.761 | 4.478 | 67.37 |  |  |  |  |  |  |
|  | 7 | 0.688 | 4.048 | 71.418 |  |  |  |  |  |  |
|  | 8 | 0.657 | 3.867 | 75.285 |  |  |  |  |  |  |
|  | 9 | 0.564 | 3.318 | 78.603 |  |  |  |  |  |  |
|  | 10 | 0.537 | 3.159 | 81.762 |  |  |  |  |  |  |
|  | 11 | 0.517 | 3.044 | 84.805 |  |  |  |  |  |  |
|  | 12 | 0.486 | 2.862 | 87.667 |  |  |  |  |  |  |
|  | 13 | 0.465 | 2.736 | 90.402 |  |  |  |  |  |  |
|  | 14 | 0.443 | 2.603 | 93.005 |  |  |  |  |  |  |
|  | 15 | 0.42 | 2.469 | 95.474 |  |  |  |  |  |  |
|  | 16 | 0.406 | 2.391 | 97.865 |  |  |  |  |  |  |
|  | 17 | 0.363 | 2.135 | 100 |  |  |  |  |  |  |
| 18 | 1 | 3.887 | 22.863 | 22.863 | 3.887 | 22.863 | 22.863 | 3.076 | 18.096 | 18.096 |
|  | 2 | 2.161 | 12.715 | 35.577 | 2.161 | 12.715 | 35.577 | 1.956 | 11.507 | 29.603 |
|  | 3 | 1.725 | 10.148 | 45.725 | 1.725 | 10.148 | 45.725 | 1.931 | 11.36 | 40.963 |
|  | 4 | 1.423 | 8.369 | 54.094 | 1.423 | 8.369 | 54.094 | 1.865 | 10.968 | 51.932 |
|  | 5 | 1.284 | 7.554 | 61.649 | 1.284 | 7.554 | 61.649 | 1.652 | 9.717 | 61.649 |
|  | 6 | 0.821 | 4.83 | 66.478 |  |  |  |  |  |  |
|  | 7 | 0.78 | 4.587 | 71.065 |  |  |  |  |  |  |
|  | 8 | 0.691 | 4.068 | 75.133 |  |  |  |  |  |  |
|  | 9 | 0.646 | 3.801 | 78.934 |  |  |  |  |  |  |
|  | 10 | 0.608 | 3.578 | 82.512 |  |  |  |  |  |  |
|  | 11 | 0.525 | 3.088 | 85.6 |  |  |  |  |  |  |
|  | 12 | 0.503 | 2.961 | 88.561 |  |  |  |  |  |  |
|  | 13 | 0.471 | 2.768 | 91.329 |  |  |  |  |  |  |
|  | 14 | 0.446 | 2.624 | 93.953 |  |  |  |  |  |  |
|  | 15 | 0.4 | 2.35 | 96.303 |  |  |  |  |  |  |
|  | 16 | 0.354 | 2.084 | 98.387 |  |  |  |  |  |  |
|  | 17 | 0.274 | 1.613 | 100 |  |  |  |  |  |  |
| Notes: Extraction method: principal component analysis. | | | | | | | | | | |

The rotated component matrix a

| Features | 16-17 element | | | | | 18element | | | | |
| --- | --- | --- | --- | --- | --- | --- | --- | --- | --- | --- |
|  | 1 | 2 | 3 | 4 | 5 | 1 | 2 | 3 | 4 | 5 |
| Parents will ask why | 0.638 | -0.134 |  | 0.127 |  | 0.754 |  |  |  |  |
| Parents encourage you to make an effort | 0.771 |  |  | 0.109 |  | 0.719 |  |  |  | 0.207 |
| Parents talk to you kindly | 0.738 | -0.118 | 0.157 |  | 0.132 | 0.624 |  |  | 0.319 |  |
| Parents encourage you to think independently | 0.751 |  |  |  | 0.145 | 0.648 |  | 0.269 | 0.119 | 0.141 |
| Parents will tell you why | 0.746 |  |  |  |  | 0.717 |  |  |  |  |
| Parents like to talk to you | 0.742 |  |  | 0.143 |  | 0.751 |  |  |  | 0.159 |
| Trust in neighbors |  | -0.168 | 0.658 | 0.187 |  | 0.138 | -0.138 |  | 0.626 |  |
| Trust in local government officials |  |  | 0.824 |  | 0.12 |  |  |  | 0.825 |  |
| Trust in doctors |  |  | 0.78 | 0.17 |  |  |  | 0.294 | 0.694 | 0.106 |
| I feel emotionally drained |  | 0.786 |  | -0.153 |  |  | 0.747 |  | -0.26 |  |
| I feel lonely | -0.114 | 0.772 |  | -0.116 | -0.145 |  | 0.782 | -0.164 |  |  |
| I feel sad and upset | -0.101 | 0.811 |  | -0.104 |  |  | 0.824 | -0.168 |  |  |
| I feel happy |  |  |  |  | 0.858 |  |  | 0.149 |  | 0.872 |
| I am happy with my life | 0.152 | -0.169 |  | 0.109 | 0.829 | 0.204 |  |  |  | 0.874 |
| Satisfaction with my life | 0.187 | -0.221 | 0.11 | 0.774 |  | 0.119 | -0.216 | 0.735 |  |  |
| Level of confidence in my future |  | -0.12 |  | 0.81 |  |  |  | 0.747 | 0.117 |  |
| How happy | 0.273 | -0.109 | 0.324 | 0.553 | 0.27 |  |  | 0.752 | 0.294 |  |
| Notes: Extraction method: principal component analysis. Rotation method: Kaiser normalized maximum variance method. a Rotation has converged after 6 iterations. | | | | | | | | | | |
